# E2F1-Associated Purine Synthesis Pathway Is a Major Component of the MET-DNA Damage Response Network

**DOI:** 10.1158/2767-9764.CRC-23-0370

**Published:** 2024-07-30

**Authors:** Michaela Poliaková Turan, Rahel Riedo, Matúš Medo, Chiara Pozzato, Manja Friese-Hamim, Jonas P. Koch, Si’Ana A. Coggins, Qun Li, Baek Kim, Joachim Albers, Daniel M. Aebersold, Nicola Zamboni, Yitzhak Zimmer, Michaela Medová

**Affiliations:** 1 Department of Radiation Oncology, Inselspital Bern University Hospital, Bern, Switzerland.; 2 Department for BioMedical Research, Radiation Oncology, University of Bern, Bern, Switzerland.; 3 Graduate School for Cellular and Biomedical Sciences, University of Bern, Bern, Switzerland.; 4 Corporate Animal Using Vendor and Vivarium Governance (SQ-AV), Corporate Sustainability, Quality, Trade Compliance (SQ), Animal Affairs (SQ-A), The Healthcare Business of Merck KGaA, Darmstadt, Germany.; 5 Center for Drug Discovery, Department of Pediatrics, Emory University School of Medicine, Atlanta, Georgia.; 6 College of Pharmacy, Kyung-Hee University, Seoul, South Korea.; 7 Research Unit Oncology, The Healthcare Business of Merck KGaA, Darmstadt, Germany.; 8 Institute of Molecular Systems Biology, ETH Zürich, Zürich, Switzerland.; 9 PHRT Swiss Multi-Omics Center, Zurich, Switzerland.

## Abstract

Various lines of investigation support a signaling interphase shared by receptor tyrosine kinases and the DNA damage response. However, the underlying network nodes and their contribution to the maintenance of DNA integrity remain unknown. We explored MET-related metabolic pathways in which interruption compromises proper resolution of DNA damage. Discovery metabolomics combined with transcriptomics identified changes in pathways relevant to DNA repair following MET inhibition (METi). METi by tepotinib was associated with the formation of γH2AX foci and with significant alterations in major metabolic circuits such as glycolysis, gluconeogenesis, and purine, pyrimidine, amino acid, and lipid metabolism. 5′-Phosphoribosyl-N-formylglycinamide, a *de novo* purine synthesis pathway metabolite, was consistently decreased in *in vitro* and *in vivo* MET-dependent models, and METi-related depletion of dNTPs was observed. METi instigated the downregulation of critical purine synthesis enzymes including phosphoribosylglycinamide formyltransferase, which catalyzes 5′-phosphoribosyl-N-formylglycinamide synthesis. Genes encoding these enzymes are regulated through E2F1, whose levels decrease upon METi in MET-driven cells and xenografts. Transient E2F1 overexpression prevented dNTP depletion and the concomitant METi-associated DNA damage in MET-driven cells. We conclude that DNA damage following METi results from dNTP reduction via downregulation of E2F1 and a consequent decline of *de novo* purine synthesis.

**Significance:**

Maintenance of genome stability prevents disease and affiliates with growth factor receptor tyrosine kinases. We identified *de novo* purine synthesis as a pathway in which key enzymatic players are regulated through MET receptor and whose depletion via MET targeting explains MET inhibition-associated formation of DNA double-strand breaks. The mechanistic importance of MET inhibition-dependent E2F1 downregulation for interference with DNA integrity has translational implications for MET-targeting-based treatment of malignancies.

## Introduction

Receptor tyrosine kinase (RTK) is a commonly expressed oncogenic protein that is associated with the onset and progression of cancers of various origins (1). Consequently, tyrosine kinase inhibitors represent an effective antitumor modality used in various clinical settings (2, 3). The RTK MET, the high-affinity receptor for hepatocyte growth factor (HGF), functions as an oncogenic driver in many distinct human tumors, including gastric, lung, brain, and head and neck cancers (4). Physiologically, MET-regulated signaling pathways function in tissue damage regeneration in adults and provide essential signals for myogenic precursor cells during embryogenesis (5). Abnormal MET receptor activation in malignancies, which results predominantly from protein overexpression with or without gene amplification (6), triggers oncogenic downstream signaling majorly through the PI3K/mTOR, STAT, and MAPK pathways, eventually enhancing tumor angiogenesis and metastasis with overall disease progression (7), and associates with poor clinical outcome (8, 9). Moreover, deregulated MET signaling has been shown to prevail as a resistance mechanism to antitumor treatment targeting other oncoproteins (10, 11). Interestingly, accumulating data imply the existence of a potential crosstalk between MET and the DNA damage response (DDR) machinery as METi results in enhanced sensitivity of MET-positive tumor models to DNA-damaging agent (DDA; refs. 12–16). METi prevents the irradiation (IR)-dependent nuclear translocation of RAD51, which is important to execute DNA double-strand break (DSB) repair via homologous recombination upon IR exposure (17). METi by its own is sufficient to increase γH2AX levels, indicating that MET signaling could have a role in DDR activation by replicative stress origin (14, 18), although the detailed molecular mechanisms underlying these observations remain largely unknown.

Molecular pathways used by tumor cells to generate energy and support biosynthesis vary from those employed by normal cells (19). Back in the 1950s, Otto Warburg postulated that malignant cells favor to break down glucose through glycolysis, even with ubiquitous oxygen to support oxidative phosphorylation (20). Importantly, because particular reprogrammed metabolic features are commonly seen across various types of cancer, altered metabolism can be considered as a driving parameter in particular malignant disorders rather than a mere passenger adaptation for cell survival and apoptotic signals (21). Furthermore, an emerging body of data indicates that intracellular circumference including cancer metabolism is tightly associated with the RTK aberrant signaling (22). It has been shown, for example, that EphA2 promotes glutamine metabolism in tumors (23), EGFR enhances aerobic glycolysis, and VEGFR2 regulates mitochondrial reprogramming that can be linked with resistance to chemotherapy-driven cell death in an acute myeloid leukemia model (24). MET targeting in tumor cells was previously shown to downregulate the TP53-induced glycolysis and apoptosis regulator with successive decrease of intracellular NADPH, leading therefore to a depletion of crucial reducing power, which is essential for the maintenance of cell survival (25). Kumar and colleagues (26) reported an autocrine loop triggered by HGF secreted by tumor-associated fibroblasts, which acts through MET to drive glycolysis in the progression of head and neck cancer.

Various reports have demonstrated that MET downstream signaling pathways impact cellular response to DNA damage and that METi induces radiosensitization (13, 17, 27, 28), raising therefore the likelihood for active involvement of this receptor in preserving genome integrity. We aimed at investigating the potential metabolic circuits downstream of MET which function within a MET-DDR signaling crosstalk. Accordingly, we conducted a metabolome-wide profiling of a panel of MET-driven cancer cells and tumor xenografts in their unperturbed state and upon METi. We show that MET signaling modulates multiple global metabolic pathways including a striking decrease in purine synthesis pathway metabolite levels caused by a depletion of enzymes involved in the *de novo* purine synthesis pathway. We demonstrate mechanistic importance of METi-dependent E2F1 downregulation for interference with DNA integrity of these cells and provide important insights into the role of METi with critical therapeutic translational implications for the treatment of malignancies through MET targeting.

## Materials and Methods

### Cell culture

The human gastric carcinoma cell lines GTL-16 (RRID:CVCL_7668) and MKN45 (RRID:CVCL_0434), SNU-638 (RRID:CVCL_0102), and KATOII and Hs746T (RRID:CVCL_0333) were kindly provided by Dr. Paolo Comoglio (Medical School University of Torino, Italy), Korean Cell Line Bank, Dr. Morag Park (Cancer Research Centre, McGill University, Montreal, Canada), and Dr. Silvia Giordano (Medical School University of Torino, Italy), respectively. The human lung carcinoma cell line EBC-1 (RRID:CVCL_U892) was obtained from Dr. Silvia Giordano (Medical School University of Torino, Italy), and lung cancer cell lines H1993 (RRID:CVCL_1512), HCC827 (RRID:CVCL_2063), H1648 (RRID:CVCL_1482), and H820 (RRID:CVCL_1592) were obtained from Dr. Sunny Zachariah (UT Southwestern Medical Center, Dallas, TX). No further authentication of these cell lines was performed.

Hs746T cell line was cultured in DMEM (GIBCO, Invitrogen) supplemented with 10% FCS (Sigma) and antibiotic-antimycotic (penicillin 100 U/mL, streptomycin sulfate 100 U/mL, amphotericin B as Fungizone 0.25 μg/mL; GIBCO). All other cell lines were cultured in RPMI medium (GIBCO, Invitrogen) supplemented with 5% to 10% FCS and antibiotic-antimycotic. NIH3T3 cell lines that stably express the M1268T or Y1248H MET-activating mutations (referred to as NIH3T3 MET M1268T and NIH3T3 MET Y1248H, respectively) were previously described, and their identities were documented by sequencing of both strands of DNA in the region of interest (29). NIH3T3 MET M1268T and Y1248H cell lines were kindly provided by Dr. Laura Schmidt (National Cancer Institute, Frederick, MD) and maintained in DMEM supplemented with 10% FCS, antibiotic-antimycotic, and 0.5 mg/mL geneticin/G-418 sulfate (GIBCO, Invitrogen).

All cells were cultured under normal oxygen conditions of 21% O_2_ and 5% CO_2_ at 37°C in a humidified incubator. All cells were regularly passaged and kept in culture for up to 2 months upon thawing to perform the described experiments.

An E2F1 or empty vector expression plasmid [pMax–E2F1 (RRID:Addgene_16007) or pMax-EV; Addgene] was transiently transfected with GTL-16 cells using Lipofectamine LTX reagent (Invitrogen) and kept until 72 hours post transfection (unless stated otherwise). GFP expression was measured to determine transfection efficiency.

### Inhibitors

MET enzymatic activity was inhibited by the selective MET tyrosine kinase small-molecule inhibitor tepotinib (MSC2156119; the Healthcare Business of Merck KGaA, Darmstadt, Germany) at a final concentration of 50 nmol/L for the indicated time points. For MAPK and PI3K inhibition, AZD6244 (Selleck Chemicals) and LY294002 (Sigma) were used, respectively, both dissolved in DMSO and used at a final concentration of 1 μmol/L.

### *In vivo* xenograft models

*In vivo* efficacy and pharmacodynamic data were generated in human lung cancer xenograft models EBC-1 and HCC827. The study designs and animal usage were approved by local animal welfare authorities (Regierungspräsidium Darmstadt, Germany, protocol registration numbers DA4/Anz.1014). For both models, 7- to 8-week-old female CD-1 nude mice (Charles River Laboratories) were used. 5 × 10^6^ tumor cells were injected subcutaneously in the flank, in 100 μL Dulbecco’s phosphate-buffered saline, tumor length (L) and width (W) were measured with calipers twice weekly, and tumor volumes were calculated using L × W2/2. When tumor xenografts reached a mean volume of 150 to 250 mm^3^, mice (*N* = 10 per treatment arm, randomized from 15 mice per arm to obtain a similar mean and median within the treatment groups) received either vehicle or tepotinib (MSC2156119). Tepotinib was formulated in 20% Kolliphor/80% of 100 mmol/L sodium acetate buffer at pH 5.5 and administered once daily via oral gavage.

### Metabolite extraction

Cells were seeded in six-well plates (each experimental condition was plated in quadruplicates) and treated as indicated. At a given time point, cells were washed with 75 mmol/L ammonium carbonate (Sigma) at pH 7.4, quenched by snap freezing with liquid nitrogen, and stored at −80°C. Metabolites were extracted with 400 μL of the extraction solution (2:2:1 acetonitrile:methanol:water; −20°C) on ice for 10 minutes. The plate was re-extracted one more time with 400 μL of extraction solution, allowing for a high recovery rate to perform qualitative comparison between conditions. The extracts were collected in a 1.5-mL microtube and centrifuged (4°C, 13,000 rpm, 2 minutes). Supernatants were stored at −20°C until analysis by MS.

For the extraction of polar metabolites from tissues, tumor tissues (20–80 mg) were fully homogenized in cold extraction solvent (70% ethanol in ddH_2_O). The samples were incubated for 1 minute in hot extraction solvent at 75°C, and the extracts were dried under vacuum and suspended in ddH_2_O. To extract polar metabolome from plasma samples, the samples were diluted 1:10 in 80% methanol and vortexed and incubated for 1 hour at 4°C, followed by centrifugation and supernatant collection.

### Nontargeted MS measurements

For nontargeted metabolite profiling, samples were analyzed by flow injection analysis on an Agilent 6550 Q-TOF instrument in negative ionization mode (m/z range of 50–1,000) at 4 GHz high-resolution mode (30). Ions were putatively annotated based on accurate mass against the KEGG (RRID:SCR_012773) database with a tolerance of 0.001 Da. Ion annotation matches are reported in Supplementary Table S1. Statistical analysis of the resulting metabolomic data was performed using custom scripts in Matlab (RRID:SCR_001622).

### Supplementation experiments

Cells were seeded in complete medium and allowed to grow for 24 hours. Afterward, the medium was changed and supplemented with either glutamine (8 mmol/L, Sigma-Aldrich), serine (1.2 mmol/L, Sigma-Aldrich), folic acid (0.008 mmol/L, Sigma-Aldrich), nucleosides (adenosine 180 mmol/L; cytidine 200 mmol/L; guanosine 170 mmol/L; thymidine 200 mmol/L; uridine 200 mmol/L; Sigma-Aldrich), or hypoxanthine (100 mmol/L, Sigma-Aldrich) at the same time point as the treatment with tepotinib.

### Delivery of irradiation

Cells were irradiated using a ^137^Cs source (Gammacell 40, MDS Nordion, Ottawa, ON, Canada) at a dose rate of 0.86 Gy/min. In all *in vitro* studies, cells were irradiated with a single dose of 10 Gy and lysed 1 hour post ionizing radiation (IR).

### Protein extraction and Western blot analysis

Cells were lysed in urea lysis buffer (HEPES, urea, sodium orthovanadate, sodium pyrophosphate, β-glycerol-phosphate) followed by sonication or in a lysis buffer composed of 2% Triton X-100, 1% Nonidet P-40, 2 mmol/L EGTA, 2 mmol/L EDTA, 300 mmol/L NaCl, 20 mmol/L Tris–HCl, 50× Na3VO4, 50× NaF, 50× ZnCl2, 50× Na2MoO4a, and a cocktail of protease inhibitors (Complete Mini, Roche) for p4E-BP1 and pMET detection. Total protein concentrations were determined with the Bio-Rad protein quantification reagent (Bio-Rad Laboratories, Inc.; RRID:SCR_008426), and 50 μg of total protein was resolved by SDS-PAGE on 7% to 12% gels. Separated proteins were transferred to PVDF membranes followed by blocking with 5% milk in TBS-Tween and overnight incubation with primary antibodies against p-Y1234/Y1235 MET (RRID:AB_331713), p-Ser473 AKT (RRID:AB_2315049), p-Thr202/Tyr204 ERK1/2 (RRID:AB_331646), p-Ser240/244 S6 (RRID:AB_331682), and p-Thr37/46 4E-BP1 (RRID:AB_560835; all Cell Signaling Technology); anti-β-actin (RRID:AB_2223041), phospho-Histone H2AX (RRID:AB_309864), and RAD51 (RRID:AB_309785; all EMD Millipore Corporation); and GART (RRID:AB_1563066) and E2F1 (RRID:AB_1793996; both Santa Cruz Biotechnology). Incubation of membranes was done with appropriate secondary antibodies (GE Healthcare), and signals were detected with ECL HRP substrate kits [Amersham Pharmacia Biotech and Thermo Fisher Scientific (#K-12045-D20)].

### Cell proliferation

Cell proliferation was determined using a resazurin sodium salt reduction assay (Sigma). Cells were seeded in a 96-well plate, and after a treatment for 72 hours, the wells were supplemented with medium containing 44 μmol/L resazurin. Resazurin reduction was colorimetrically measured 1 and 6 hours later (570/600 nm) with a Tecan Reader (Tecan Group Ltd.). Results were normalized to vehicle-treated controls and represented as a mean of at least three independent experiments.

### Crystal violet assay

Cells were plated at low confluency either in 24-well plates (8,000 cells/well) or in 48-well plates (3,000 cells/well) in triplicates. Twenty-four hours after seeding, the cells were treated with the indicated doses of tepotinib and cisplatin [Selleckchem (#S1166)] either alone or in combination and were left to proliferate for 72 hours posttreatment. Relative cell number was measured by crystal violet staining (0.1% in 20% methanol) of adherent cells after 10 minutes of fixation in 4% paraformaldehyde. After washing twice and air-drying, stained cells were decolored with 5% acetic acid, and OD600 was measured with a spectrophotometer. Data were analyzed with GraphPad Software. Graphical data are shown as mean ± SD of technical replicates (dots). Statistical analyses were done using one-way ANOVA.

### Apoptosis assay

Caspase-3 enzymatic activity was determined via a fluorogenic assay based on the caspase-3-specific substrate Ac-DEVD-AMC (Calbiochem). Cells were seeded in 10-cm plates; the substrate was added to cell lysates after 72 hours of treatment. Fluorescence was measured at excitation and emission wavelengths of 380 and 460 nm, respectively, with an Infinite 200 plate reader (Tecan Group Ltd.). Caspase-3 activity was normalized to protein content, and the results shown are representative of at least three independent experiments.

### Real-time PCR

Total RNA was extracted from 80% confluent cell cultures using Trizol lysis according to the manufacturer’s instructions (Roche). Reverse transcription of mRNA was performed using the Omniscript RT Kit (Qiagen). Quantitative PCR of PRPS1, PPAT, GART, E2F1, E2F2, RRM1, and RRM2 was performed using a 7900HT fast real-time PCR system with a TaqMan assay (Applied Biosystems). The mean CT was determined from triplicate experiments and mRNA levels normalized to those obtained for 28S ribosomal protein. Changes in expression were determined by calculations of ∆∆CT.

### Fluorescent microscopy

Cells were plated in eight-chamber slides (4 × 10^4^ cells/chamber) 24 hours before treatment and/or transfection. Cells were fixed with 4% formaldehyde, washed with TBS, permeabilized with 0.25% Triton-X100, and washed with TBS. Afterward, cells were blocked with a 3% goat serum. Cells were incubated with phospho-Histone H2AX antibody (1:1,000, Millipore Corporation; RRID:AB_309864) and E2F1 antibody (1:50, Santa Cruz Biotechnology; RRID:AB_1793996) overnight at 4°C and washed with TBS-Tween. Afterward, cell were labeled with secondary antibodies (1:200; Alexa Fluor antibodies, Abcam), washed with TBS-Tween, and later incubated with ′4′-6′-diamidino-2-phenylindole (DAPI; Sigma-Aldrich). Cells were mounted with VECTASHIELD Antifade Mounting Medium (Vector Laboratories) and analyzed with Leica DMI4000. Images were processed using ImageJ (RRID:SCR_003070). Positive γH2AX foci per cell were counted (30 cells evaluated per condition).

### Immunohistochemistry

Xenograft tumors were fixed in 10% neutral buffered formalin at room temperature for 24 hours, transferred to 70% ethanol, and then embedded in paraffin. After cutting, the sections were deparaffinized and rehydrated. The heat-induced antigen retrieval was achieved in citrate buffer (pH 6). Subsequently, peroxidase blocking and serum blocking were performed. The sections were stained with the primary antibodies for pMET (1:150, Tyr1234/1235, clone D26, Cell Signaling Technology; RRID:AB_2143884), E2F-1 (1:50, Cell Signaling Technology; RRID:AB_2096936), and GART (1:50, clone SC66-02, Invitrogen; RRID:AB_2809520) and were detected by the Vectastain ABC Kit (Vector Laboratories) and 3,3'-diaminobenzidine (DAB; Sigma-Aldrich). Hematoxylin was used for counterstaining, and the slides were scanned with a Pannoramic 250 Flash III Scanner (3DHISTECH). The percentage of positively stained area was quantified with ImageJ. Statistical analysis was performed using Prism 8 (GraphPad; RRID:SCR_002798). *P* values <0.05 were considered significant (^∗^, *P* < 0.05; ^∗∗^, *P* < 0.01; ^∗∗∗^, *P* < 0.001; ^∗∗∗∗^, *P* < 0.0001).

### dNTP measurements

GTL-16 and EBC-1 cells were seeded at a density of 2 × 10^6^ cells and treated with either DMSO or tepotinib for 24 hours (if not indicated otherwise). After treatment, the cells were washed with PBS and lysed in ice-cold 65% (vol/vol) methanol. The cells were scraped and counted for the normalization; the pellet was vortexed vigorously and incubated at 95°C for 3 minutes. After centrifugation (3 minutes at 14 K RPM), the pellets were discarded, and the samples consisting of methanol solution were speed vacuumed until they dried. Subsequent dNTP measurements were performed as described previously (31).

### Transcriptomics

Nucleic acids were extracted following lysis of cell pellets in RTL lysis buffer (AllPrep DNA/RNA/miRNA Universal Kit, Qiagen). The lysates were homogenized in CK14 Precellys homogenization tubes (Labgene Scientific) using the Minilys homogenizer (Bertin Technologies). RNA was purified using a robotic workstation (QIAcube, Qiagen), and their quality was assessed by the Fragment Analyzer (Advanced Analytical Technologies, Inc.). RNA sequencing libraries were prepared using the Illumina TruSeq Stranded Total RNA reagents (Cat. nr. RS-122-2201; Illumina) according to the protocol supplied by the manufacturer and using 750 ng of total RNA.

Samples were processed with SureSelect Library Prep Kit (Agilent). Results of the differential expression analysis of the transcriptomic data are provided in Supplementary Table S2.

### The Cancer Genome Atlas (TCGA) data analysis

We used the data portal of the NCI Genomic Data Commons (https://portal.gdc.cancer.gov/ RRID:SCR_014514) and its API to gather data on lung, stomach, colorectal, and head and neck cancer cases. We obtained the basic case information, corresponding clinical files, and transcriptome profiling files. Normalization of the raw gene counts in the transcriptomic data was done by the calcNormFactors function of the R package edgeR (version 3.16.5). Basic statistics of the analyzed TCGA datasets is provided in Supplementary Table S3. Samples with low and high gene expression are chosen based on percentiles of expression values for a given tumor site and tissue type (e.g., low expression for all samples below the 20th percentile and high for all samples above the 80th percentile).

### Statistical analysis

Statistical analysis and graphical presentation of the data were performed using Prism Graph (version 7.04). Data for each treatment group are represented as means ± SEM as indicated and compared with evaluate the significance using the Student *t* test. Differences with *P* values <0.1 were considered statistically significant (^∗^, *P* < 0.1; ^∗∗^, *P* < 0.01; ^∗∗∗^, *P* < 0.001, ^∗∗∗∗^).

### Ethics statement

The study designs and animal usage were approved by local animal welfare authorities (Regierungspräsidium Darmstadt, Germany, protocol registration numbers DA4/Anz.1014).

### Data availability

The data generated in this study are available within the article and its supplementary data files or upon request from the corresponding author.

## Results

### Markers of metabolic activity and DNA damage in MET-positive models following receptor targeting

To investigate the metabolic circuits affected by MET RTK activity and its targeted inhibition, a panel of 10 cellular models of MET-positive cell lines including seven MET-driven (GTL-16, MKN45, KATOII, SNU638, Hs746T, EBC-1, H1993) and three non-MET-driven cancer cell lines (H1648, H820, and HCC827) has been used. All MET-driven lines harbor amplification of the MET gene (32, 33) and hence display MET addiction. In addition, isogenic NIH3T3 cells that ectopically express drug-sensitive MET-activating point mutation M1268T or drug-resistant activating point mutation Y1248H MET-mutated variants (denoted as “M1268T cells” and “Y1248H cells,” respectively) were employed (34, 35). MET enzymatic activity was inhibited by the MET tyrosine kinase small-molecule inhibitor tepotinib (36).

**Figure 1:**
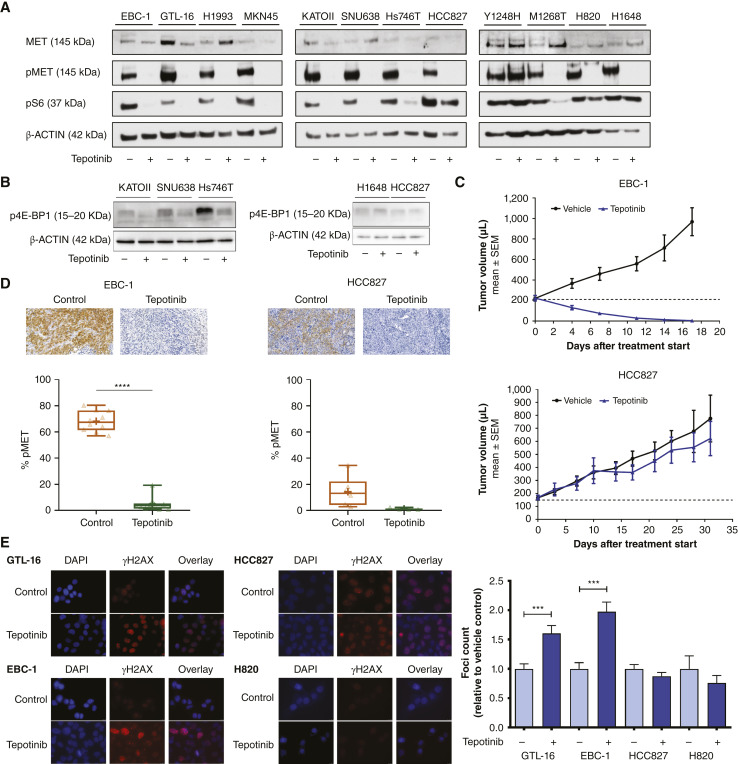
Responses of a panel of MET-positive MET-driven and non-MET-driven human cancer cell models to selective MET targeting. **A,** Whole-cell lysates were subjected to Western blotting using specific antibodies against MET, pMET, and pS6 following the treatment either with vehicle or 50 nmol/L tepotinib (EMD1214063, shortly EMD) for 24 hours. Western blots representative of *N* = 3 independent experiments are shown. **B,** Whole-cell lysates were subjected to Western blotting using a specific antibody against p4E-BP1 following the treatment either with vehicle or 50 nmol/L tepotinib (EMD1214063, shortly EMD) for 24 hours. Western blots representative of *N* = 3 independent experiments are shown. **C,** EBC-1 (top) and HCC827 (bottom) xenograft tumor volume upon vehicle or METi treatment (*N* = 10). **D,** pMET Tyr1234/5 expression in EBC-1 (left) and HCC827 (right) tumor xenografts treated by vehicle or METi (sampling was performed 2 hours posttreatment). Representative images (top) and quantification of pMET-positive area (bottom) are provided [EBC-1: *N* = 10 (control), *N* = 10 (tepotinib); HCC827: *N* = 6 (control), *N* = 4 (tepotinib); unpaired Student *t* test, *, *P* < 0.05; **, *P* < 0.01; ***, *P* < 0.001; ****, *P* < 0.0001]. **E,** Representative images (left) of nuclear foci of γH2AX in MET-driven GTL-16 and EBC-1 and non-MET-driven HCC827 and H820 cells. Untreated or METi-treated (50 nmol/L) cells were fixed; γH2AX-positive foci were visualized by confocal microscopy and counted (right), *N* = 3, unpaired Student *t* test, *, *P* < 0.05; **, *P* < 0.01; ***, *P* < 0.001; ****, *P* < 0.0001.

All the cell lines in the panel express constitutively active MET receptor as inferred from considerable basal MET autophosphorylation levels on Tyr1234/5 (Fig. 1A). Likewise, tepotinib effectively decreases MET autophosphorylation (pMET) in all these cells except in the tepotinib-resistant Y1248H cell line (Fig. 1A). To assess if the inhibition of the MET receptor is further transmitted to inhibition of its downstream signaling and associated with metabolic pathways, we evaluated the impact of METi by tepotinib on phosphorylation of the ribosomal S6 protein on Ser-240/244 as a surrogate of mTORC1 activity known to promote cellular metabolism (37). S6 phosphorylation has been previously extensively correlated with various metabolic aspects including glucose homeostasis, and genetically modified mice that express S6 mutated at the C-terminus serines 235/236/240/244/247 display various traits associated with growth and end energy deficits (38). Here, the METi-triggered drop in pMET levels translated into a decrease in phosphorylated S6 (pS6) protein levels in METi-responsive cell lines GTL-16, MKN45, KATOII, SNU638, Hs746T, EBC-1, and H1993 as well as in the drug-sensitive M1268T MET-mutated cell line. In these cells, the inhibition of MET signaling impacts cell proliferation and later leads to cell death. Alternatively, the MET-positive but non-MET-dependent cell lines H1648, H820, and HCC827 do respond to METi in terms of MET autophosphorylation inhibition, while no pS6 decrease can be observed (Fig. 1A), thus advocating the possibility that these cells do not require MET signaling for their growth and survival. Lastly, as the Y1248H cell line does not respond to MET targeting by MET small-molecule inhibitors, also pS6 protein levels are not affected by METi (Fig. 1A). Depending on the cell line and treatment conditions, METi has a similar impact also on the phosphorylation levels of the eukaryotic translation initiation factor 4E-binding protein (4E-BP1), another mTORC1 effector (Fig. 1B; Supplementary Fig. S1). These differential responses to METi are likewise reflected *in vivo*, in which METi treatment leads to a complete EBC-1 tumor regression, whereas the growth of the HCC827 tumors is not affected by METi (Fig. 1C). Analogous to the *in vitro* findings, both these xenograft models feature considerable basal MET Tyr1234/5 phosphorylation levels that are efficiently blocked by METi (Fig. 1D). To further correlate these differences between MET-driven and non-MET-driven cells with a DDR phenotype, we studied the extent of DNA DSBs- in cells treated with tepotinib by evaluating the presence of Ser139-phosphorylated histone variant H2AX (γH2AX) in two MET-driven (GTL-16 and EBC-1) and two non-MET-driven (HCC827 and H820) cells. METi treatment alone resulted in γH2AX foci in MET-driven GTL-16 and EBC-1 but not in the non-MET-driven HCC827 and H820 cells (Fig. 1E). These findings support our previously published data suggesting that MET-DDR crosstalk is characteristic only for a particular subset of cancers with active MET receptor signaling (28, 39).

### Impact of METi on global metabolome in MET-positive models

We have further used this heterogeneous panel of cell lines expressing active MET receptor to study the impact of METi and its downstream signaling on the global changes in cellular metabolome. For metabolomic analysis, extracts under the effect of 50 nmol/L of the inhibitor tepotinib upon 24 hours were profiled by LC/MS-MS. Principal component analysis performed to evaluate the qualitative differences between treatment conditions and cell lines revealed only moderate dissimilarities, with no evident separation between treated and nontreated MET-driven cell lines (Supplementary Fig. S2A). However, non-MET-driven cell lines H1648 and HCC827 group together, whereas MET-driven cell lines EBC-1, GTL-16, KATOII, MKN45, and SNU-638 cluster together with the non-MET-driven cell line H820.

To build on our previous data, we aimed to investigate how MET targeting impacts cellular metabolism and its relation to phenotypic changes. We observed apparent differences on the metabolic level between the two groups of MET-driven and non-MET-driven lines upon 24 hours of exposure to 50 nmol/L of the MET inhibitor tepotinib. As illustrated by the volcano plots in Supplementary Fig. S2B, which provide an overall score for the changed metabolism upon tepotinib exposure, statistically significant differences (|log_2_FC| > 0.5; adj. *P* value < 0.01) occurred exclusively in the MET-driven cell line models. Interestingly, the MET-mutated drug-resistant variant Y1248H displayed some alterations upon inhibitor administration, suggesting that these changes may be related to the off-target effects of the drug (Supplementary Fig. S2B).

**Figure 2:**
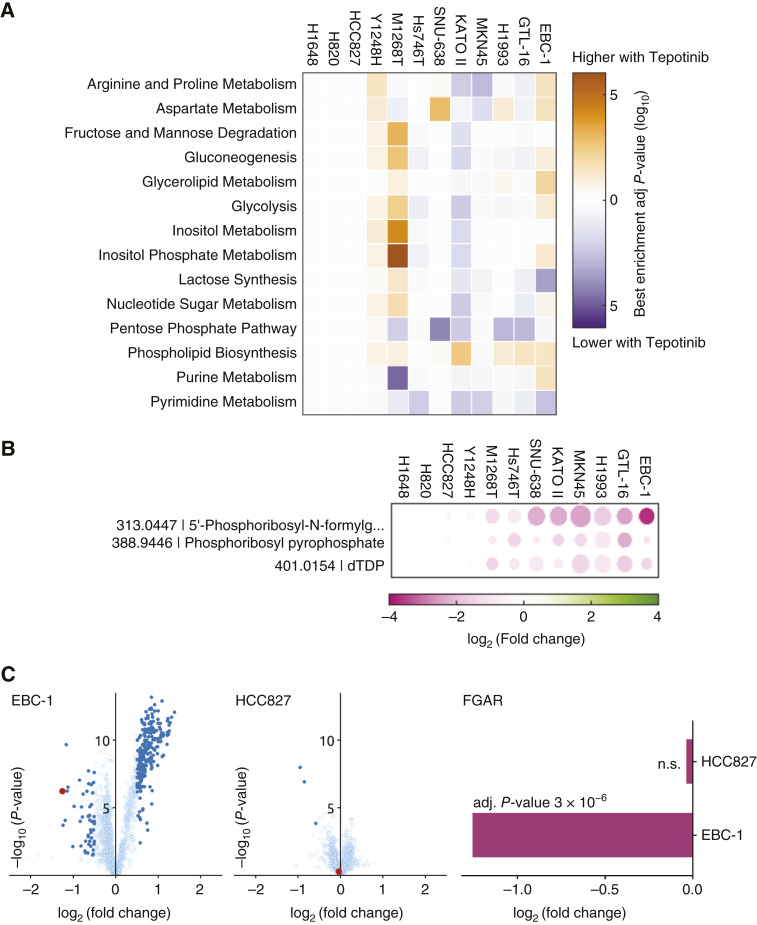
Metabolic changes in MET-driven and non-MET-driven cancer cells and xenografts. **A,** Pathway enrichment analysis upon MET inhibition (*N* = 3, |log_2_FC| > 0.5; adj. *P* value < 0.01) revealed alterations observed in 12 MET-expressing cell lines upon 24 hours of METi. **B,** Changes in the abundance of the three metabolite ions [5′-phosphoribosyl-N-formylglycinamide (FGAR), phosphoribosyl pyrophosphate (PRPP), and deoxythymidine diphosphate (dTDP)] that change most upon METi in MET-driven cell lines. Symbol sizes represent the adjusted *P* values from 1 (smallest) to 10^−8^ (largest) using a log scale. No significant changes in these ions occurred in the non-MET-driven cell lines H1648, H820, HCC827, and Y1248H. **C,** Left, Differences in metabolite ion (symbols) abundance in METi- and vehicle-treated tumor xenografts. Symbols corresponding to statistically significant differences (|log_2_FC| > 0.5; *P* value < 0.01) are indicated in dark blue, and FGAR is marked in red. Right, Changes in the FGAR metabolite ion abundance in HCC827 and EBC-1 tumor xenografts upon METi.

Pathway enrichment analysis (Fig. 2A; |log_2_FC| > 0.5; adj. *P* value < 0.01) highlighted significant metabolic pathway perturbations that are associated with METi in MET-driven cell lines. These alterations pertain to amino acid-related pathways such as aspartate, arginine, and proline metabolism (up to a threefold alteration in METi-treated cell lines compared with controls); glycolysis, gluconeogenesis, and pentose phosphate pathway (PPP; a two- to fourfold change); purine and pyrimidine metabolism (a two- to sixfold change); and lipid- and inositol-related metabolism (a two- to sixfold change). Importantly, no significant changes about the activity of metabolic pathways were detected in the non-MET-driven cellular models (Fig. 2A).

Next, we investigated which exclusive metabolites were significantly and commonly altered upon METi across all MET-driven cell lines. The many changes in metabolite ions that occurred in these cells relate, for example, to a decrease in TCA cycle intermediates (malate, fumarate, and succinate) or an increase in distinct lipid-related metabolite ions (e.g., glycerophosphocholine and glycerol phosphate; Supplementary Fig. S2B and S2C). Among all alterations, three metabolites were significantly and consistently decreased in all eight MET-driven cellular systems but not in non-MET-driven cell lines: 5′- phosphoribosyl-N-formylglycinamide (FGAR), phosphoribosyl pyrophosphate (PRPP), and deoxythymidine diphosphate (dTDP; Fig. 2B). Interestingly, these three metabolites all relate to the *de novo* purine synthesis pathway. The most significantly altered metabolite in the group of the eight MET-driven cell lines was FGAR, with a METi-induced log_2_ fold change of up to −3 (corresponding to an almost 90% relative decrease), depending on a particular cell line (Fig. 2B). Similar effects can be observed also *in vivo* in which METi leads to significant metabolic changes as well as to FGAR ion decrease in EBC-1 but not in HCC827 xenografts (Fig. 2C). These findings prompted us to investigate further the role of FGAR and *de novo* purine synthesis in MET signaling and its crosstalk with DDR.

### Assessment of the state of the purine synthesis pathway upon METi

Purine levels in human cells are maintained by a regulated activity of the salvage and *de novo* biosynthetic pathways. The *de novo* purine biosynthetic axis is an energy-intensive pathway that produces inosine 5′-monophosphate (IMP) from phosphoribosyl pyrophosphate (PRPP). The entire pathway consists of 10 steps catalyzed by six enzymes (40). To study if the decrease in FGAR following METi is a passenger observation or a driver metabolic event, we performed a series of interventions that intend to answer this question. Specifically, we aimed at plausibly rescuing MET-driven cells following METi via either supplementing the cells with glutamine, serine, and folic acid (Supplementary Fig. S3A and S3B), the building blocks of the purine synthesis pathway, by supplementation with hypoxanthine and nucleosides (Supplementary Fig. S4A and S4B), the metabolites used by the salvage pathway, or by deoxyribonucleotide triphosphate (dNTP) supplementation. To that end, the two MET-driven cell lines GTL-16 and EBC-1 were treated by METi along with each of these supplements to avoid METi-mediated reduction in proliferation and increase in apoptosis in these cells (14, 34). However, no changes in proliferation (Supplementary Figs. S3A and S4A) and no profound decrease in apoptosis (Supplementary Figs. S3B and S4B) could be detected upon any of these supplementations in combination with METi.

**Figure 3:**
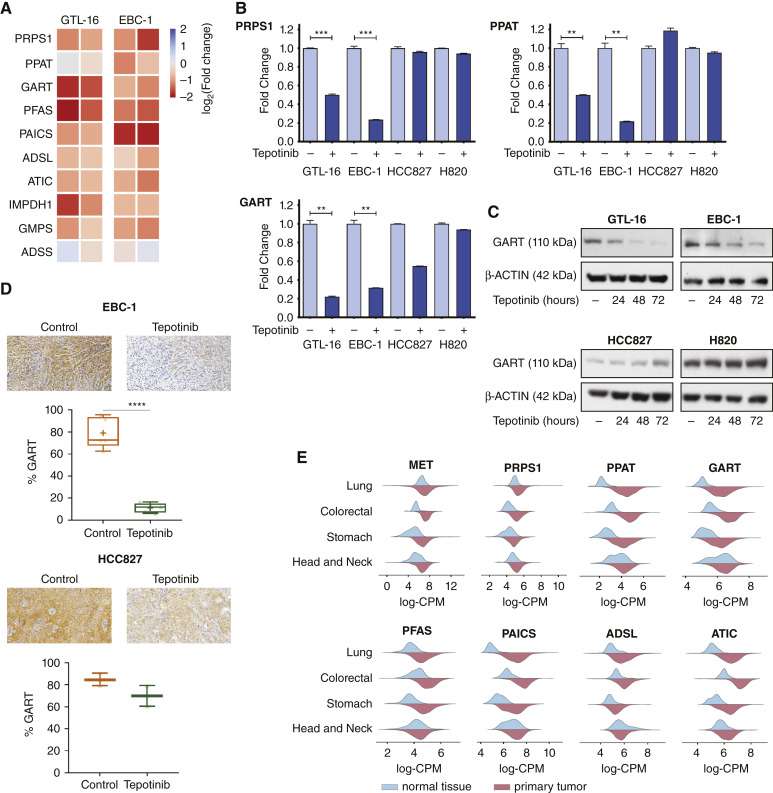
Impact of MET inhibition on the purine synthesis pathway. **A,** Transcript levels of purine synthesis enzymes are downregulated upon 24 hours of METi for EBC-1 and GTL-16 cells (*N* = 2). **B,** mRNA levels of PRPS1, PPAT, and GART upon METi in MET-driven (EBC-1 and GTL-16) and non-MET-driven (HCC821 and H820) cell lines assessed by quantitative real-time PCR (*N* = 3, unpaired Student *t* test, *, *P* < 0.05; **, *P* < 0.01; ***, *P* < 0.001; ****, *P* < 0.0001). **C,** Whole-cell lysates were subjected to Western blotting using specific antibodies against GART following treatment either with vehicle or 50 nmol/L tepotinib for 24, 48, and 72 hours in MET-driven (EBC-1 and GTL-16) and non-MET-driven (HCC821 and H820) cell lines. Representative images of *N* = 3 independent experiments are shown. **D,** GART expression levels in EBC-1 (top) and HCC827 (bottom) tumor xenografts treated by vehicle or METi. Representative images (top) and quantification of GART-positive area (bottom) are provided [(EBC-1: *N* = 5 (control), *N* = 5 (tepotinib); HCC827: *N* = 3 (control), *N* = 2 (tepotinib); unpaired Student *t* test, *, *P* < 0.05; **, *P* < 0.01; ***, *P* < 0.001; ****, *P* < 0.0001)]. **E,** MET, PRPS1, PPAT, GART, PFAS, PAICS, ADSL, and ATIC transcript distribution in normal tissue vs. primary tumor in lung, colorectal, stomach, and head and neck cancer TCGA datasets.

As the attempts to rescue the cells by supplementations did not turn out to be successful, we hypothesized that the METi-related drop of the purine metabolite FGAR might be profoundly related to a depletion of enzymes that drive the purine synthesis pathway. Consequently, we performed complementary transcriptomics of these two MET-driven cellular models upon METi. This analysis revealed that all crucial purine biosynthesis enzymes (e.g., PRPS1, PPAT, GART, PFAS, PAICS, ADSL, ATIC, IMPDH1, GMPS, ADSS) are downregulated upon METi on the mRNA level (Fig. 3A). The impact of METi on mRNA levels of PRPS1, PPAT and GART, three enzymes preceding FGAR synthesis, was verified by quantitative real-time PCR in both MET-driven and non-MET-driven cells. We could validate that their mRNA expression indeed decreases following METi exclusively in the MET-driven EBC-1 and GTL-16 cells, whereas no change in mRNA levels of PRPS1, PPAT, and GART was seen upon METi in non-MET-driven cell lines HCC827 and H820 (Fig. 3B). GART catalyzes three distinct steps of the *de novo* purine synthesis pathway, including the formation of FGAR (41), the most significant hit in our metabolomic analysis. Importantly, the decrease in GART mRNA levels detected 24 hours post METi treatment initialization was followed by a decrease of its total protein levels following prolonged METi (48 and 72 hours; Fig. 3C). GART protein levels decreased upon METi solely in MET-driven but not in the non-MET-driven cellular systems in both *in vitro* and *in vivo* settings (Fig. 3C and 3D).

To gain translational insights into the potential relevance of the *de novo* purine synthesis pathway in human cancer, TCGA data revealed that the mRNA levels of purine synthesis enzymes are commonly upregulated in primary tumors in patients with gastric, lung, colorectal, and head and neck cancers at the transcriptomic level as compared with their normal tissue counterparts (Fig. 3E). Moreover, underlying the plausible importance of MET in onset and/or progression of these cancer types, our TCGA data analysis shows that also MET mRNA levels are increased in these cancer types as compared with the normal tissue mRNA expression levels (Fig. 3E).

### METi induces depletion of dNTPs via hindered production of ribonucleotide reductase

**Figure 4:**
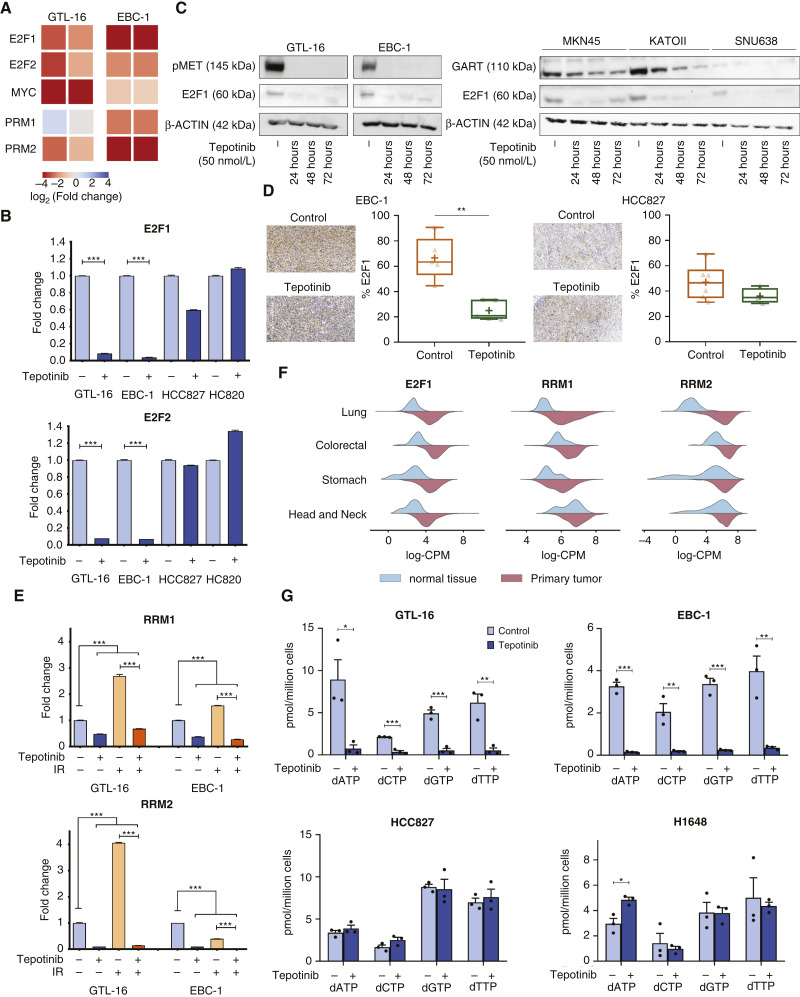
Transcriptional effectors involved in regulation of purine synthesis pathway. **A,** Complementary transcriptomic analysis of purine synthesis pathway regulation effectors in EBC-1 and GTL-16 cells. MYC, E2F1, E2F2, and both large (RRM1) and small (RRM2) ribonucleotide reductase subunits were downregulated upon METi in MET-driven EBC-1 and GTL-16 cell lines (*N* = 2). Differences between the gene’s expression (METi vs. control) are shown. **B,** Quantitative real-time PCR assessment of mRNA levels of E2F-1 and E2F-2 upon METi in MET-driven (EBC-1 and GTL-16) and non-MET-driven (HCC821 and H820) cell lines (*N* = 3, unpaired Student *t* test, *, *P* < 0.05; **, *P* < 0.01; ***, *P* < 0.001; ****, *P* < 0.0001). **C,** Whole-cell lysates of MET-driven cell lines EBC-1 and GTL-16 were subjected to Western blotting using pMET (Tyr1234/5) and E2F1 antibodies following their treatment with vehicle or 50 nmol/L tepotinib for 24, 48, and 72 hours (left). Protein levels of GART and E2F1 were assessed by Western blotting in MET-driven MKN45, KATOII, and SNU-638 cells (right). ß-Actin was used as a loading control. Representative images of *N* = 3 independent experiments are shown. **D,** E2F1 expression in EBC-1 (left) and HCC827 (right) tumor xenografts treated by vehicle or METi. Representative images (left) and quantification of E2F1-positive area (right) are provided [EBC-1: *N* = 5 (control), *N* = 5 (tepotinib); HCC827: *N* = 6 (control), *N* = 4 (tepotinib); unpaired Student *t* test, *, *P* < 0.05; **, *P* < 0.01; ***, *P* < 0.001; ****, *P* < 0.0001]. **E,** mRNA levels of RRM1 and RRM2 were assessed by real-time PCR following METi (24 hours, 50 nmol/L), irradiation (10 Gy, 1-hour time-point post IR), and upon combinatory treatment in GTL-16 and EBC-1 cells (*N* = 3, unpaired Student *t* test, *, *P* < 0.05; **, *P* < 0.01; ***, *P* < 0.001; ****, *P* < 0.0001). **F,** Comparisons of E2F1, RRM1, and RRM2 transcript distribution in normal tissue vs. primary tumor in lung, colorectal, stomach, and head and neck cancer TCGA datasets. **G,** dNTP levels in MET-driven cell lines GTL-16 and EBC-1 and non-MET-driven cell lines HCC827 and H1648 in response to METi by tepotinib (24 hours, 50 nmol/L), *N* = 3, unpaired Student *t* test, (*, *P* < 0.05; **, *P* < 0.01; ***, *P* < 0.001; ****, *P* < 0.0001).

Numerous critical transcription factors, particularly MYC and E2F family members E2F-1 and E2F-2, regulate genes encoding enzymes involved in nucleotide synthesis (42). Indeed, our transcriptomic datasets revealed that these three transcription factors are all downregulated in MET-driven EBC-1 and GTL-16 cells upon METi (Fig. 4A). As MYC drives tumorigenesis by enhancing a chaotic state of flux of every biochemical pathway and E2F family was shown to be transcriptionally activated by MYC (43), we restricted our further investigations to E2F members as an approximation of transcriptional regulations of the studied pathways. Real-time PCR confirmed the decrease of E2F1 and E2F2 mRNAs following METi in both cell lines EBC-1 and GTL-16, whereas no change in their transcripts was observed in the non-MET-driven cell lines HCC827 and H820 (Fig. 4B). As E2F1 in particular has been proposed as a major regulator of nucleotide biosynthesis (42), we have further followed on its protein expression. The drop of the E2F1 mRNA was associated with the decrease of its total protein levels exclusively in MET-driven cell lines and their derived xenografts (Fig. 4C and D).

E2F1 was also reported to regulate the transcription of ribonucleotide reductase (RNR; ref. 44), an essential enzyme that catalyzes the generation of dNTPs from ribonucleotides, products of the purine pathway (45). Thus, we next explored the changes in levels of RRM1 and RRM2, the large and small subunits of RNR, respectively. RRM2 transcripts were strongly downregulated in both GTL-16 and EBC-1 cells following METi, whereas the mRNA of the RRM1 subunit seems to decrease especially in EBC-1 cells (Fig. 4A). To validate the transcriptomic data, real-time PCR was employed, and RRM1 and RRM2 mRNA levels were assessed in cells treated by METi alone and in combination with a single dose of IR (10 Gy, lysis 16 hours post IR). This combinatory treatment was used as a control for RNR activity because RNR levels were shown to increase following DNA damage when cellular DNA must be efficiently repaired, and adequate levels of dNTPs are needed (46). Interestingly, mRNA levels of both RRM1 and RRM2 decreased following 24 hours of METi in both GTL-16 and EBC-1 cells as compared with untreated controls. In response to IR, transcript levels of both RNR subunits increased as expected (Fig. 4E; ref. 46). However, the combined treatment of METi and irradiation decreased mRNA levels of both RRM1 and RRM2 subunits (Fig. 4E). Analogous changes were induced by these treatments also when assessing mRNA levels of GART and E2F1 (Supplementary Fig. S5).

We hypothesize that the impact of METi on RNR synthesis may lead in combination with external DNA damage elicited by IR to an impaired supply of *de novo* dNTPs necessary for radiation-induced DNA repair, increasing therefore the radiosensitivity of the cells. These data also suggest a novel mechanism plausibly explaining the previously reported DNA damage induced by a prolonged METi treatment (14). Interestingly from a clinical perspective, analysis of data available from the TCGA database indicates that mRNA levels of E2F1 as well as of both RNR subunits are upregulated in primary tumors in patients with gastric, lung, colorectal, and head and neck cancers as compared with their normal tissue levels (Fig. 4F).

In parallel to evaluating RNR expression pattern upon METi, we studied dNTP levels in the corresponding cells, as in addition to DNA replication and repair fidelity, an increase in the dNTP pool is fundamental also during the S phase for cell proliferation (47, 48). Indeed, as the results in Fig. 4G show, METi induced the depletion of dNTPs (dATP, dCTP, dGTP, and dTTP) in MET-driven cell lines EBC-1 and GTL-16, whereas no change in their levels was observed in non-MET-driven cell lines HCC827 and H1648.

### E2F1 downregulation upon METi is linked to METi-dependent DNA damage

Within the frame of the transcriptomic analysis and subsequent confirmation by real-time PCR, we observed that METi results in a nearly fourfold decrease of the transcription factor E2F1 exclusively in METi-responsive cell lines EBC-1 and GTL-16 (Fig. 4A). A fundamental role of E2F1 biology is its involvement in both cell cycle regulation and apoptosis. Hence, our findings prompted us to investigate potential MET downstream signaling pathways that may link the receptor to E2F1 and whether the diminished levels of E2F1 could be associated with the reduction in purine synthesis enzymes GART and RNR, the observed dNTP depletion, and the increased γH2AX levels.

**Figure 5:**
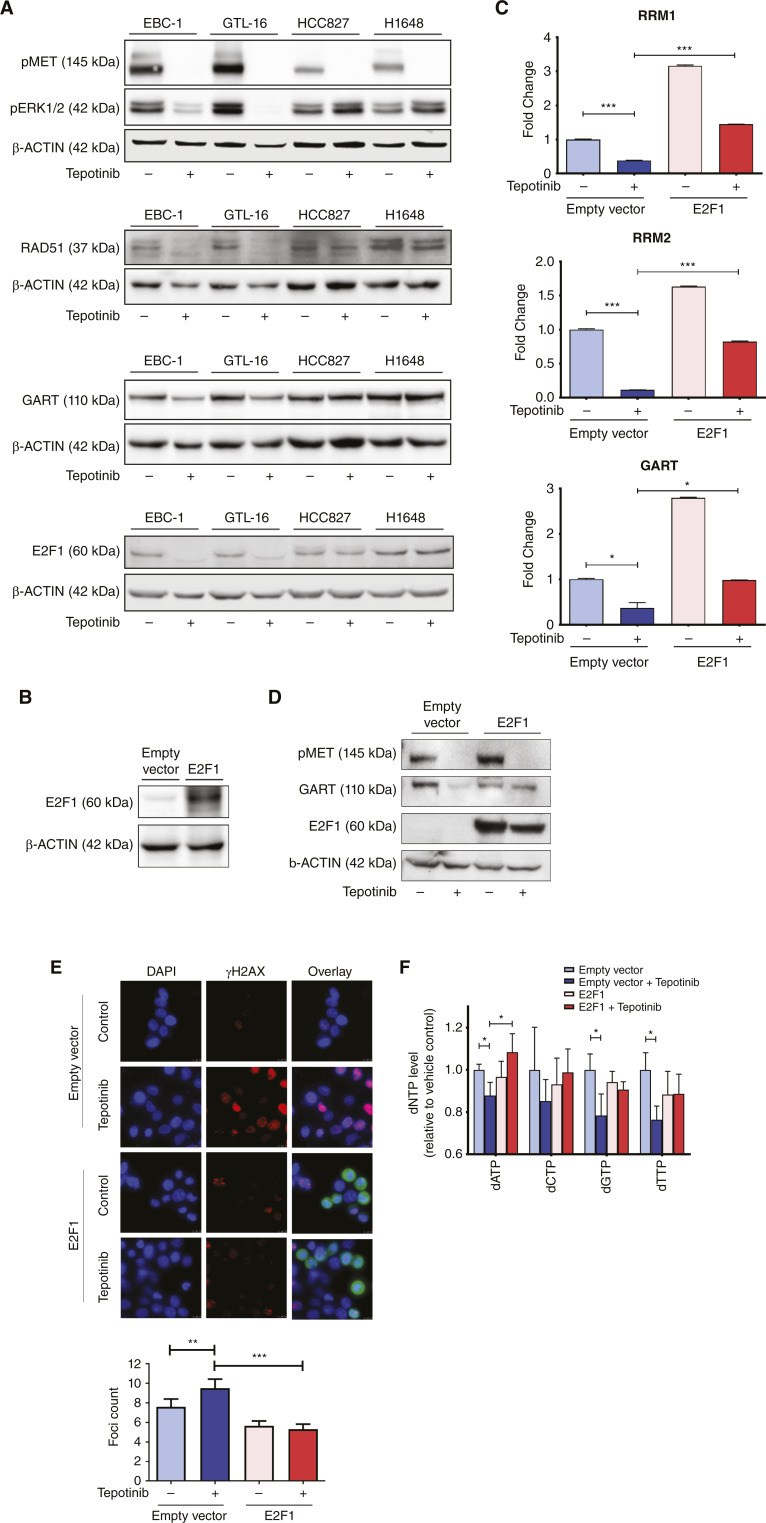
METi-dependent DNA damage induction mediated by E2F1 downregulation. **A,** Whole-cell lysates of MET-driven (GTL-16 and EBC-1) and non-MET-driven (HCC827 and H1648) cells were subjected to Western blotting using specific antibodies against pMET, pERK1/2, RAD51, GART, and E2F1 following treatment with vehicle or 50 nmol/L tepotinib for 24 (pMET, pERK1/2, and RAD51) or 48 (GART and E2F1) hours. Lysates for pMET and pERK1/2 detection were prepared by using the NP-40 lysis buffer, and lysates for detection of the other proteins were prepared by using the urea lysis buffer. ß-Actin was used as a loading control. Representative images of *N* = 3 independent experiments are shown. **B,** Whole-cell lysates were subjected to Western blotting using specific antibody against E2F1 in untreated empty vector and E2F1-overexpessing GTL-16 cells (72 hours post transfection). ß-Actin was used as a loading control. Representative images of *N* = 3 independent experiments are shown. **C,** mRNA levels of RRM1, RRM2, and GART following E2F1 overexpression in GTL-16 (72 hours post transfection and 48 hours post tepotinib treatment) as assessed by quantitative real-time PCR (*N* = 3, unpaired Student *t* test, *, *P* < 0.05; **, *P* < 0.01; ***, *P* < 0.001; ****, *P* < 0.0001). **D,** Whole-cell lysates were subjected to Western blotting using specific antibodies against pMET, GART, and E2F1 following indicated treatments in E2F1-overexpessing cell line GTL-16 (72 hours post transfection and 48 hours post tepotinib treatment). ß-Actin was used as a loading control. Representative images of *N* = 3 independent experiments are shown. **E,** Representative images of nuclear γH2AX foci in perturbed (tepotinib 50 nmol/L, 8 hours) and unperturbed E2F1-overexpressing (16 hours post transfection) GTL-16 cells and in empty vector control cells (top). Bottom, γH2AX foci count per cell (blue: DAPI; red: γH2AX; green: E2F1-overexpressing cells), *N* = 3, unpaired Student *t* test, *, *P* < 0.05; **, *P* < 0.01; ***, *P* < 0.001; ****, *P* < 0.0001. **F,** dNTP levels in MET-driven E2F1-overexpressing GTL-16 cells in response to METi (50 nmol/L, 8 hours). Cells were collected 16 hours post transfection [*N* = 3, unpaired Student *t* test, (*, *P* < 0.05; **, *P* < 0.01; ***, *P* < 0.001; ****, *P* < 0.0001)].

As E2F1 activity that promotes GART synthesis was shown to depend on MAPK activation (49), we aimed to test if this or the other major MET downstream signaling pathway, the PI3K cascade, is involved in the observed METi-dependent E2F1 modulation. Thus, we assessed GART protein levels in GTL-16 and EBC-1 cells after blocking either the ERK/MAPK (MEK inhibition) or the PI3K/AKT (PI3K inhibition) pathways (Supplementary Fig. S6). Indeed, while treatment by the MEK inhibitor AZD6244 recapitulated the effect of tepotinib and E2F1-transcriptionally regulated GART protein levels decreased, inhibition of the PI3K by LY294002 did not affect GART expression (Supplementary Fig. S6). These data suggest that the observed METi-induced drop in E2F1 levels and subsequent reduction in purine synthesis enzyme abundance are events mediated by the ERK/MAPK pathway. We further confirmed that the treatment with tepotinib downregulates the MAPK/ERK pathway in MET-driven cells by systematically studying the activation of this pathway. We demonstrated the decrease in ERK1/2 phosphorylation, E2F1, and GART expression, as well as the expression of the RAD51 recombinase, a DDR protein and one of the plethora of genes regulated by E2F1 (Fig. 5A; ref. 50).

To confirm the direct impact of METi-dependent E2F1 depletion on purine synthesis, we transiently expressed E2F1 in MET-driven GTL-16 cancer cells (Fig. 5B). In cells that ectopically overexpress E2F1, METi does not lead to the downregulation of E2F1 transcriptional targets RRM1, RRM2, and GART on both mRNA (Fig. 5C) and protein (Fig. 5D) levels as in the empty vector-transfected control cells.

Furthermore, we investigated the impact of E2F1 overexpression on the previously documented ability of METi to induce DNA damage by evaluating γH2AX foci formation in E2F1 transiently overexpressing GTL-16 cells. Transient overexpression of E2F1 abrogated the METi-induced increase in γH2AX foci formation (Fig. 5E). This finding highlights a possibly important role for E2F1 in either prevention of DNA damage formation or efficient execution of DNA DSB repair by MET-expressing cancer cells following METi reported previously. To further investigate the E2F1-related abrogation of the METi-associated elevation in γH2AX levels and demonstrate that the lack of accumulation of γH2AX is possibly a result of the shortage of dNTPs that disable efficient DNA repair of endogenously occurring DNA DSBs, we evaluated the dNTP levels in the corresponding cells. Indeed, our data show that whereas METi caused an overall decrease in dNTPs in the empty vector-transfected cells, E2F1 transiently overexpressing cells abolished the drop of dNTPs upon the introduction of the MET inhibitor (Fig. 5F), confirming therefore the importance of the E2F1-purine pathway to METi-associated DNA DSBs.

### Co-expression of MET and associated purine synthesis pathway genes in patients’ samples and therapeutic implications

**Figure 6:**
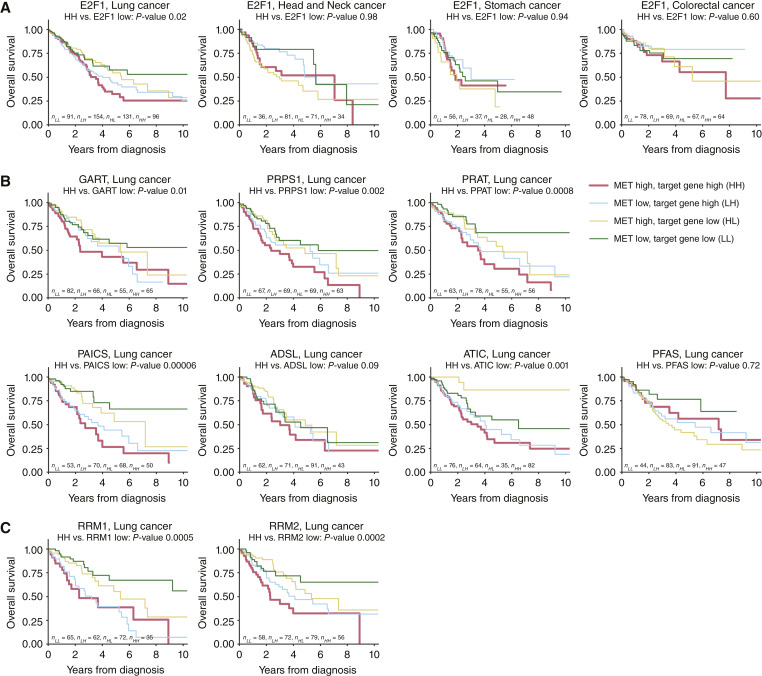
Survival analysis focused on the MET-E2F1-purine synthesis axis in clinical samples of TCGA cancer cohorts. Samples are differentiated by the under- and overexpression of individual transcripts. **A,** Impact of E2F1 and MET mRNA co-expression on survival of patients with lung, head and neck, stomach, and colorectal cancers. MET low/high percentiles 33/67. **B,** Impact of the mRNA co-expression of MET and GART/purine synthesis pathway-related enzymes on survival of patients with lung cancer. MET low/high percentiles 25/75. **C,** Impact of MET and RNR subunit (RRM1 and RRM2) mRNA co-expression on survival of patients with lung cancer. MET low/high percentiles 25/75.

Based on our experimental findings and to assess the translational relevance of the MET-E2F1-purine synthesis network described in this study, we obtained from the TCGA database gene expression (RNAseq) and clinical data for four distinct cancer types featuring MET expression. We found that patients with lung cancer with high tumoral expression of E2F1 and MET have significantly worse survival as compared with patients with low expression of E2F1 (Fig. 6A; significance analysis is provided in Supplementary Table S4). For head and neck, stomach, and colorectal cancer cases, co-high expression of E2F1 and MET did not significantly impact survival (Fig. 6A). This finding is not surprising as high MET tumoral expression does not necessarily reflect the role of MET as a driving oncogene and the frequency of MET as a driver varies between cancer types (51, 52). For example, whereas lung cancers are very often MET driven caused by the presence of MET exon 14 skipping mutations (53, 54), MET-overexpressing head and neck squamous cell carcinomas are MET driven much more rarely (55, 56). Thus, a more refined stratification of clinical datasets would be possibly required to uncover the correlation of the MET-E2F1 axis with survival in these other cancer types.

To explore the interplay between MET, E2F1, and purine synthesis in clinical cases of lung cancer, we stratified them based on different percentiles of high and low gene expression into groups of low (25th percentile) and high (75th percentile) expression. We found that the co-high expression of MET and purine synthesis genes leads to worse survival as compared with the set of tumors with low GART expression (Fig. 6B) for five purine synthesis genes (GART, PRPS1, PPAT, PAICS, and ATIC) out of seven considered. Additionally, for ADSL, there is a tendency for worse survival (significance analysis results are provided in Supplementary Table S4). Moreover, when assessing transcript levels of the two RNR subunits, patients with co-high expression of MET and RRM1 or RRM2 had significantly worse survival compared with patients with low expression of RNR subunits (Fig. 6C; Supplementary Table S4).

**Figure 7:**
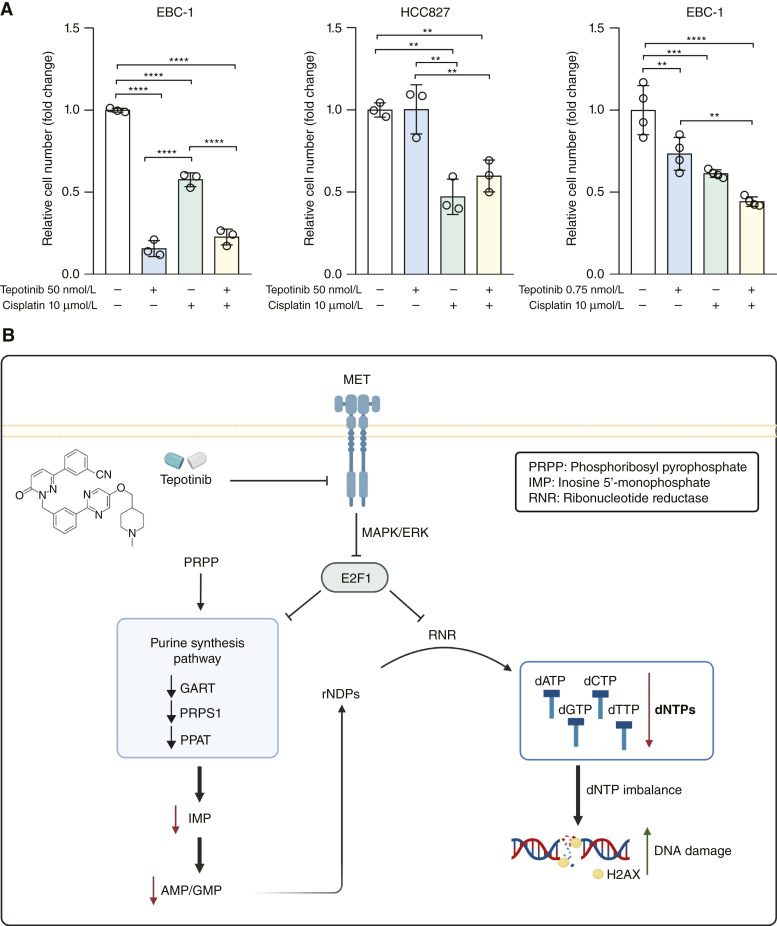
Therapeutic implications of the MET-E2F1-purine synthesis pathway as a component of the MET-DDR crosstalk. **A,** Proliferation of EBC-1 and HCC827 cells upon treatments with the indicated doses of tepotinib, cisplatin, and their combination assessed by crystal violet assay 72 hours posttreatment. Data points are represented as mean ± SD of technical replicates (dots). Statistical analyses were done using one-way ANOVA. **B,** Schematic representation of the proposed MET-E2F1-purine synthesis pathway and its interplay with the DDR in MET-driven cancer cells. MET inhibition by the small-molecule inhibitor tepotinib downregulates E2F1 via the MAPK pathway, consequently downregulating purine synthesis enzymes (e.g., GART) and RNR. This leads to the depletion of dNTPs, compromising the ability of cells to repair DNA damage. Created with BioRender.com.

Although these results are because of the lack of patient cohorts treated by METi rather of a descriptive nature, the correlations demonstrate that the MET-E2F1-purine synthesis cascade is frequently altered in human cancer, and one of the tasks of the upcoming clinical trials should be to elucidate the role of this axis in responses to anti-MET targeted-monotherapies and combined treatment regimens. Preclinically, numerous studies demonstrated the advantage of METi in combination with chemotherapy (57). The rationale behind the enhanced efficacy of such combinations stems from the notion that MET can protect cancer cells from the DNA damage induced by chemotherapeutic drugs (58, 59). We assessed cell proliferation upon the combination of METi by 50 nmol/L tepotinib with cisplatin in MET-driven and non-MET-driven cell lines EBC-1 and HCC827, respectively. As shown in Fig. 7A, METi dramatically decreased the proliferation of the EBC-1 cells with and without cisplatin, whereas no impact of METi could be seen in the HCC827 cell line. Thus, in this setting, the combination treatment did not show any advantage on either of the two cell lines compared with single treatments. However, with a substantial decrease in the concentration of tepotinib in the EBC-1 cell line, the combination of METi with cisplatin had a greater effect compared with single treatments (Fig. 7A). At least two implications of these observations should be considered when translating the findings of this and similar studies to therapy designs: only MET-driven cancers, identified, for example, by the activation of the here-reported MET-E2F1-purine synthesis cascade (Fig. 7B), respond to MET targeting via small-molecule inhibitors, and METi can sensitize them to chemotherapies pending protocol optimizations.

## Discussion

The MET RTK operates as an oncogenic driver in distinct types of cancers, such as non-small cell lung cancer and gastrointestinal carcinomas (60, 61). Moreover, the MET/HGF pathway is a key determinant of various resistance mechanisms to targeted therapies (11). Additionally, targeting of MET similarly to other RTKs, such as EGFR, IGF1R, and VEGFR, increases tumor cytotoxicity to DDAs (18). Here, we examined the impact of MET signaling inhibition by the highly selective and potent anti-MET small-molecule inhibitor tepotinib (62) in MET-driven gastric and lung cancer cell lines and *in vivo* xenografts and report that tepotinib sensitizes cancer cells to IR by depleting the *de novo* purine synthesis pathway and dNTPs via E2F1 axis *in vitro*.

It is not yet fully understood what molecular mechanisms underlie the crosstalk between MET and DDR and if this link is affiliated with specific metabolic pathways. Here, we first observed that METi by tepotinib was by itself not only sufficient to inhibit MET downstream signaling (Fig. 1A and 1B) but also to augment γH2AX levels in GTL-16 and EBC-1 cells (Fig. 1C), indicating the generation of DSBs as reported also previously using another anti-MET small-molecule PHA665752 (14). It has been proposed that MET is actively involved in the repair of damage induced by exogenous sources as well as in the repair of DNA lesions generated under physiologic conditions such as oxidative stress (17).

The metabolomic analysis demonstrated that a specific metabolite, FGAR, decreased upon METi in all METi-responsive cells and xenografts (Fig. 2B and 2C). FGAR is a metabolite of the purine synthesis pathway in which the key enzymes involved in this axis represent promising anticancer therapeutic targets, because the biosynthesis of nucleotides is critical for rapidly proliferating cancer cells (63). We report that METi decreased the FGAR levels via the drop of mRNA and protein levels of GART and additional enzymes participating in purine synthesis (Fig. 3B and 3C). Similar results were obtained using EGFRi-responsive cell lines HCC827 and PC9 treated by EGFR inhibitors gefitinib and erlotinib, in which another branch of the nucleotide biosynthesis, the enzymes of the pyrimidine biosynthesis pathway, was dependent on EGFR signaling (64).

Nucleotides are essential precursors for activated forms of carbohydrates and lipids, which participate in numerous vital cellular functions such as DNA replication and RNA transcription (42). The nucleotide metabolic gene promoters are enriched with E2F1 sites, and, notably, MYC regulates E2F1 as a direct target, thus together coordinating the nucleotide metabolism (65). As an important transcription factor, E2F1 is involved in a specific expression of RNR, a rate-limiting enzyme in DNA nucleoside metabolism (42). In recent years, many studies have focused on the significance of E2F1 and RNR in response to DNA damage. DNA damage-induced RNR expression increased dNTP pools, and its overexpression may account for radioresistance (66). It has been reported that E2F1 activity contributes to cell survival and promotes the repair of UV-induced DNA damage (67). Moreover, either E2F1 knockdown or a deactivating mutation hindered the activation of NBS1, the accumulation of RAD51, and the formation of DNA foci containing RAD51, RPA, and NBS1 (68). DNA repair was demonstrated to be considerably reduced and delayed, and the formation of γH2AX foci increased upon reduced E2F1 following DNA damage (68). Noteworthy, we observed a drop in E2F1 mRNA and protein levels following the inhibition of the MET receptor (Fig. 4B–4D). Analogously, a study conducted by Nikolai and colleagues (69) demonstrated that a growth factor receptor HER2 regulates E2F1 to coordinate cell cycle progression and DNA replication.

An important aspect of DDR is the induction of the so-called metabolic rewiring to advocate the resolution of extrinsic and intrinsic genotoxic stress. A major mechanism upon which cell metabolism can regulate DNA damage is via upregulation of the nucleotide pools by enzyme enhancement (70). Further, a few studies have focused on the importance of E2F1 and RNR, having a role in DDR. Kunos and colleagues (46) reported that the inhibition of RNR with a chemotherapeutic radiosensitizer 3-AP resulted in prolonged radiation-induced DNA damage and extended G1/S phase cell cycle arrest, consequently increasing the radiation sensitivity of human cancers. Castillo and colleagues (71) described an induction of E2F1 following DNA damage, resulting in promoting DNA repair, reducing apoptotic response, and increasing cell survival capability to maintain the genome integrity. Interestingly, our results revealed an upregulation of RNR subunits and the purine synthesis enzymes GART and E2F1 after irradiation, and strikingly, this upregulation was blocked upon co-treatment with a METi (Fig. 4E; Supplementary Fig. S5). This further supports the notion of aberrant MET signaling as a target for radiosensitization.

A critical factor in sustaining genome stability is the establishment of dNTP levels which is achieved in part by RNR activity (72). dNTPs are essential for both DNA replication and repair (72), and their depletion leads to a global inhibition of DNA replication and fork stalling (73), thus compromising the ability of irradiated cells to repair their DNA. Interestingly, our results show dropped dNTP levels following METi in MET-driven cells (Fig. 4G), thus compromising the ability of irradiated cells to repair their DNA.

To elucidate the mechanism by which METi induces the downregulation of E2F1-transcriptionally regulated GART, Graves and colleagues (74) demonstrated that the MAPK cascade regulates *de novo* pyrimidine biosynthesis. Similarly, we observed a downregulation in GART protein levels upon inhibition of the MAPK axis with the MEK1/2 inhibitor AZD6244 (Supplementary Fig. S6).

Our current data suggest that MET signaling-dependent E2F1 downregulation underlies the reported METi-induced DNA damage phenotype. Transient E2F1 overexpression in METi-responsive cell line GTL-16 restored dNTP levels and led to the increase of purine synthesis protein GART and RNR subunits, resulting in bypassed γH2AX foci formation induced by METi alone. Overall, the depleted levels of E2F1 and its regulated machinery caused by METi indicate the compromised ability of irradiated cells to repair their DNA.

Based on these data, we propose that the previously reported METi-mediated radiosensitization occurs via METi-induced dNTP depletion led by the downregulation of the E2F1 transcription factor and consequent reduction of purine synthesis enzymes (e.g., GART and PPAT) and RNR (Fig. 7B). Analysis of expression levels of key actors of the proposed MET-E2F1-purine synthesis interplay in clinical samples available in the TCGA database and their association with patients’ survival indicates the plausible utility of these assessments for patient stratification. Furthermore, the here-identified interference with DNA synthesis as one potential mechanism of METi-induced cell death could provide a basis for exploring new combinations of therapies directed at E2F1, GART, or RNR, as well as resistance mechanisms of MET-targeting therapies. In this sense, MYC amplification was recently identified as a potential negative predictor of response to METi tepotinib in patients with advanced NSCLC with high-level MET amplification (75). Although this needs to be exploited in future studies, the observed MYC amplification in these tumors might counteract the METi-induced and E2F1-dependent reduction in purine synthesis.

## Supplementary Material

Figure S1 p4E-BP1 protein levels upon MET inhibition: Whole-cell lysates were subjected to Western blotting using a specific antibody against p4E-BP1 following the treatment either with vehicle or 50nM tepotinib (EMD1214063, shortly EMD) for either 4 or 24h as indicated. Western blots representative of *N*=3 independent experiments are shown.

Figure S2 Suppementary metabolomics data: (A) Heat map determining top hits of metabolite ions in all cell lines at 24 hrs time-point post METi (|log2FC| > 0.5; adj.*P* value < 0.01). (B) Differences in metabolite ions (dots) abundance in METi-treated (24 hrs) and control cells. Statistically significant differences (|log2FC| > 0.5; *P* value < 0.01) are highlighted dark blue. (C) Enrichment analysis of the metabolomics data for GTL-16 and EBC-1 cells after 50 nM METi for 24 hrs, focusing on purine-related pathways.

Figure S3 Supplementations with glutamine, serine, and folic acid: (A) Proliferation of GTL-16 and EBC-1 cells upon supplementation with glutamine, serine or folic acid. (B) Apoptosis (caspase-3 activation) in GTL-16 and EBC-1 cells upon supplementation with glutamine, serine or folic acid.

Figure S4 Supplementations with nucleosides and hypoxanthine: (A) Proliferation of GTL-16 and EBC-1 cells upon supplementation with nucleosides or hypoxanthine. (B) Apoptosis (caspase-3 activation) in GTL-16 and EBC-1 upon supplementation with nucleosides, hypoxanthine or their combination.

Figure S5 GART and E2F1 mRNA levels following METi combined with IR: mRNA levels of GART (left) and E2F1 (right) following METi (50nM, 24 hr), IR (10 Gy, 1 hr) and their combination in GTL-16 and EBC-1 cells.

Figure S6 GART protein expression upon MAPK and PI3K targeting: GART protein levels in untreated GTL-16 and EBC-1 cells and after MAPK (AZD6244) and PI3K (LY294002) pathways inhibition. ß Actin was used as a loading control.

Table S1 Differential abundance analysis and ion annotation matches for non-targeted metabolomics measurements.

Table S2 Differential expression analysis of the GTL-16 and EBC-1 transcriptomics data.

Table S3 Basic statistics of the downloaded TCGA data.

Table S4 Summary of the significance analysis for the survival analyses.
