# Supplementary material for: E2F1-Associated Purine Synthesis Pathway Is a Major Component of the MET-DNA Damage Response Network
Source: Cancer Res Commun. 2024 Jul 30;4(7):1863–80. doi: 10.1158/2767-9764.CRC-23-0370 (PMC11288008; doi:10.1158/2767-9764.CRC-23-0370)
Supplement: Figure S1 — p4E-BP1 protein levels upon MET inhibition: Whole-cell lysates were subjected to Western blotting using a specific antibody against p4E-BP1 following the treatment either with vehicle or 50nM tepotinib (EMD1214063, shortly EMD) for either 4 or 24h as indicated. Western blots representative of N=3 independent experiments are shown. [file crc-23-0370_figure_s1_supps1.pdf]

Supplementary Figure 1

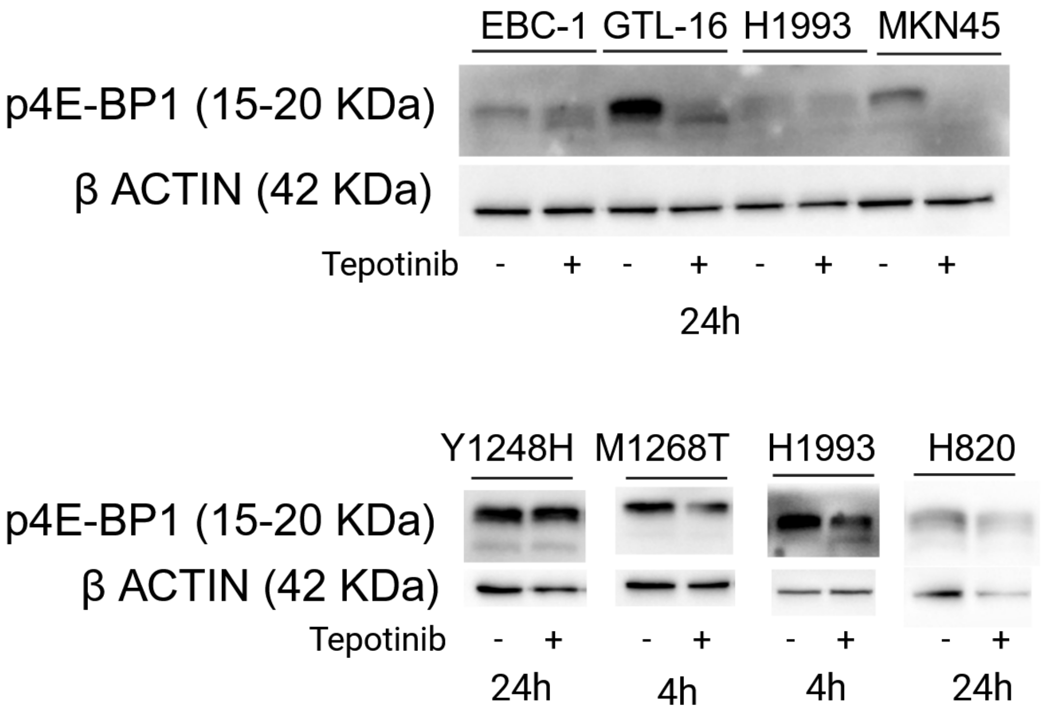

Supplementary Figure 1: p4E-BP1 protein levels upon MET inhibition

Whole-cell lysates were subjected to Western blotting using a specific antibody against p4E-BP1 following the treatment with either vehicle or 50nM tepotinib (EMD1214063, shortly EMD) for either 4 or 24h as indicated. Western blots representative of N=3 independent experiments are shown.
