# Supplementary material for: E2F1-Associated Purine Synthesis Pathway Is a Major Component of the MET-DNA Damage Response Network
Source: Cancer Res Commun. 2024 Jul 30;4(7):1863–80. doi: 10.1158/2767-9764.CRC-23-0370 (PMC11288008; doi:10.1158/2767-9764.CRC-23-0370)
Supplement: Figure S2 — Suppementary metabolomics data: (A) Heat map determining top hits of metabolite ions in all cell lines at 24 hrs time-point post METi (|log2FC| > 0.5; adj.P value < 0.01). (B) Differences in metabolite ions (dots) abundance in METi-treated (24 hrs) and control cells. Statistically significant differences (|log2FC| > 0.5; P value < 0.01) are highlighted dark blue. (C) Enrichment analysis of the metabolomics data for GTL-16 and EBC-1 cells after 50 nM METi for 24 hrs, focusing on purine-related pathways. [file crc-23-0370_figure_s2_supps2.pdf]

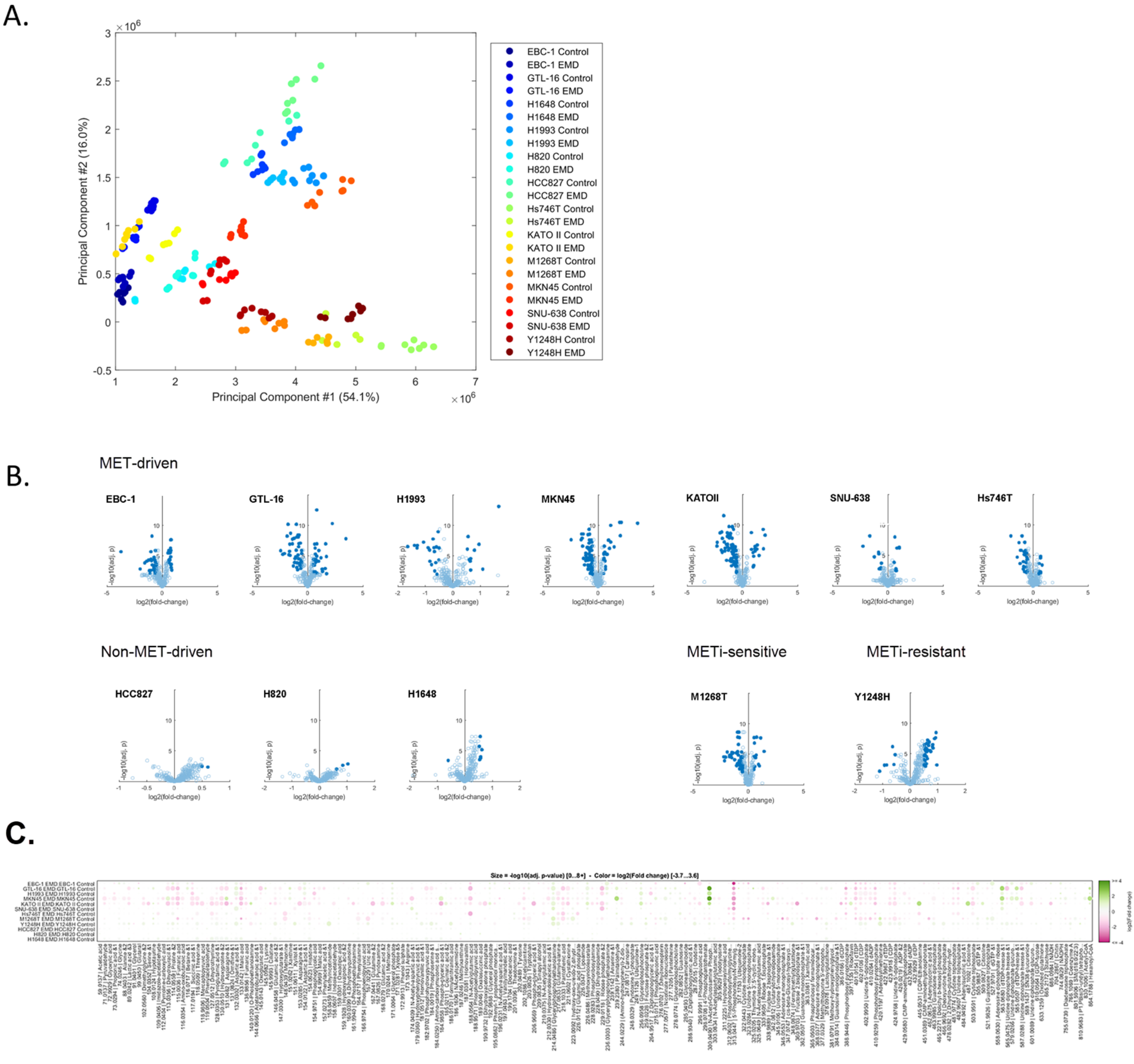

Supplementary figure 2

Supplementary Figure 2: Supplementary metabolomics data

A. Heat map determining top hits of metabolite ions in all cell lines at 24 hrs time-point post METi (|log2FC| > 0.5; adj.p-value < 0.01).
B. Differences in metabolite ions (dots) abundance in METi-treated (24 hrs) and control cells. Statistically significant differences (|log2FC| > 0.5; p-value < 0.01) are highlighted dark blue.
C. Enrichment analysis of the metabolomics data for GTL-16 and EBC-1 cells after 50 nM METi for 24 hrs, focusing on purine-related pathways.
