# Supplementary material for: E2F1-Associated Purine Synthesis Pathway Is a Major Component of the MET-DNA Damage Response Network
Source: Cancer Res Commun. 2024 Jul 30;4(7):1863–80. doi: 10.1158/2767-9764.CRC-23-0370 (PMC11288008; doi:10.1158/2767-9764.CRC-23-0370)
Supplement: Figure S3 — Supplementations with glutamine, serine, and folic acid: (A) Proliferation of GTL-16 and EBC-1 cells upon supplementation with glutamine, serine or folic acid. (B) Apoptosis (caspase-3 activation) in GTL-16 and EBC-1 cells upon supplementation with glutamine, serine or folic acid. [file crc-23-0370_figure_s3_supps3.pdf]

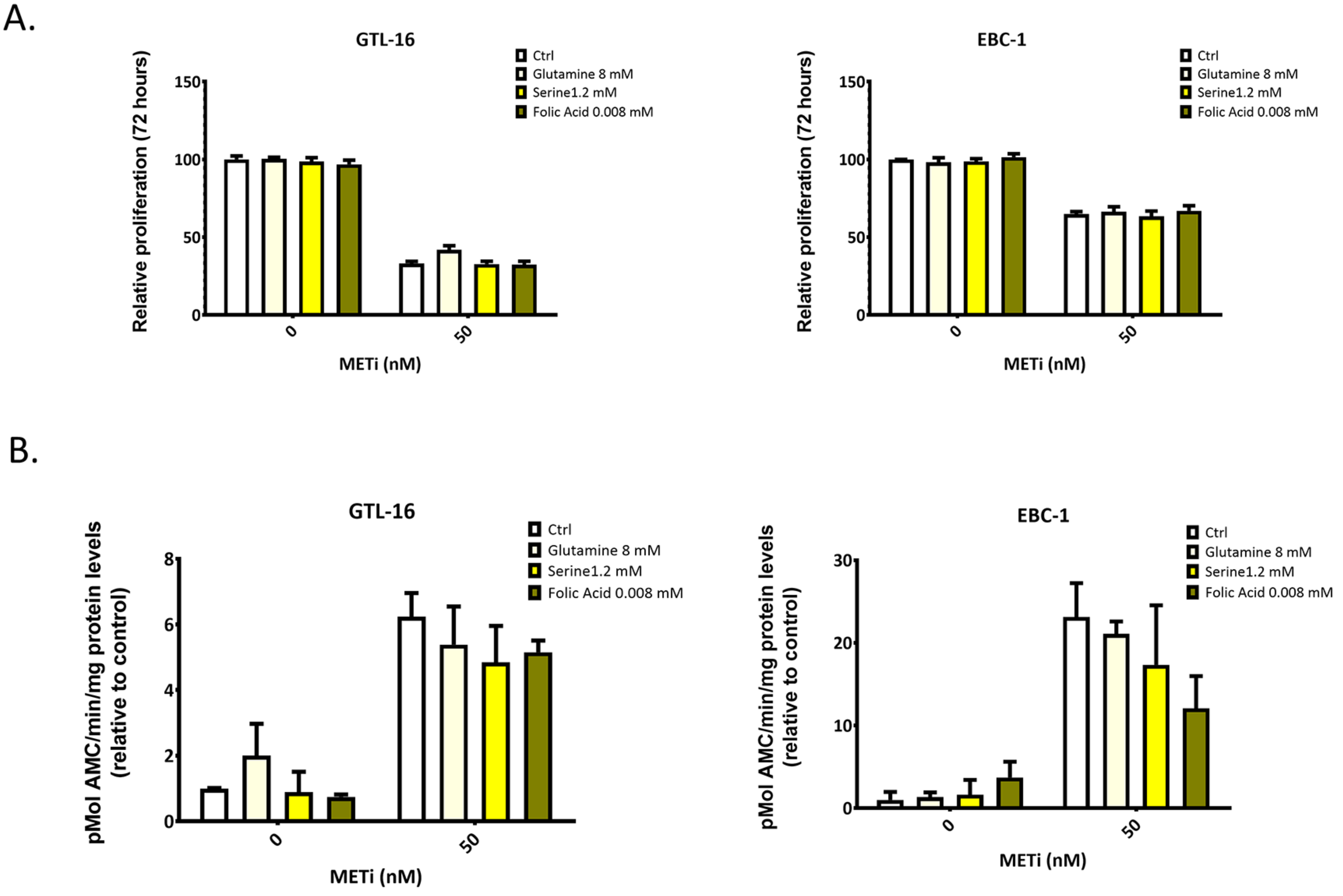

Supplementary figure 3

Supplementary Figure 3: Supplementations with glutamine, serine, and folic acid

A. Proliferation of GTL-16 and EBC-1 cells upon supplementation with glutamine, serine or folic acid.
B. Apoptosis (caspase-3 activation) in GTL-16 and EBC-1 cells upon supplementation with glutamine, serine or folic acid.
