# Supplementary material for: E2F1-Associated Purine Synthesis Pathway Is a Major Component of the MET-DNA Damage Response Network
Source: Cancer Res Commun. 2024 Jul 30;4(7):1863–80. doi: 10.1158/2767-9764.CRC-23-0370 (PMC11288008; doi:10.1158/2767-9764.CRC-23-0370)
Supplement: Figure S4 — Supplementations with nucleosides and hypoxanthine: (A) Proliferation of GTL-16 and EBC-1 cells upon supplementation with nucleosides or hypoxanthine. (B) Apoptosis (caspase-3 activation) in GTL-16 and EBC-1 upon supplementation with nucleosides, hypoxanthine or their combination. [file crc-23-0370_figure_s4_supps4.pdf]

A.

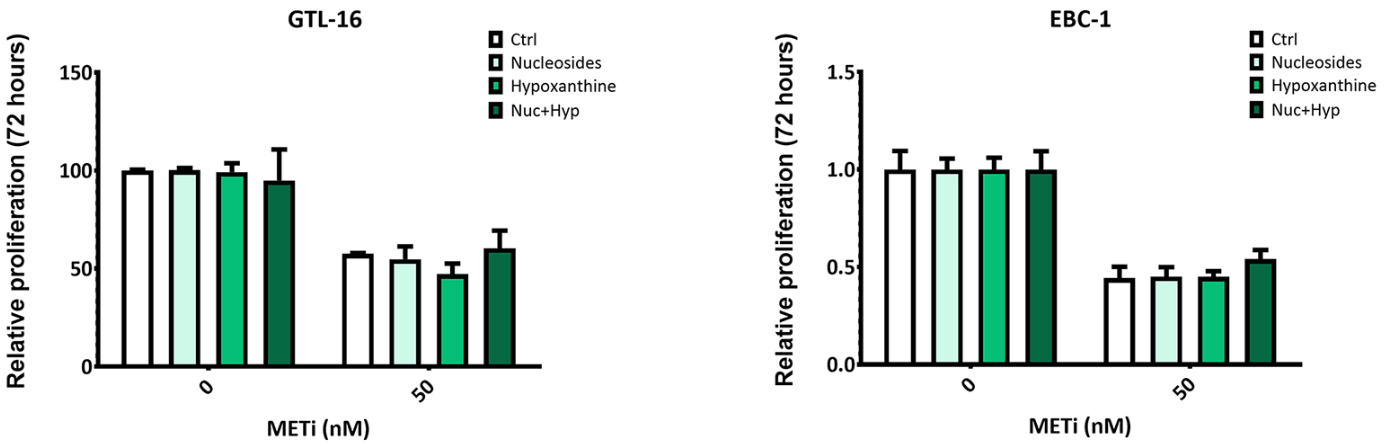

B.

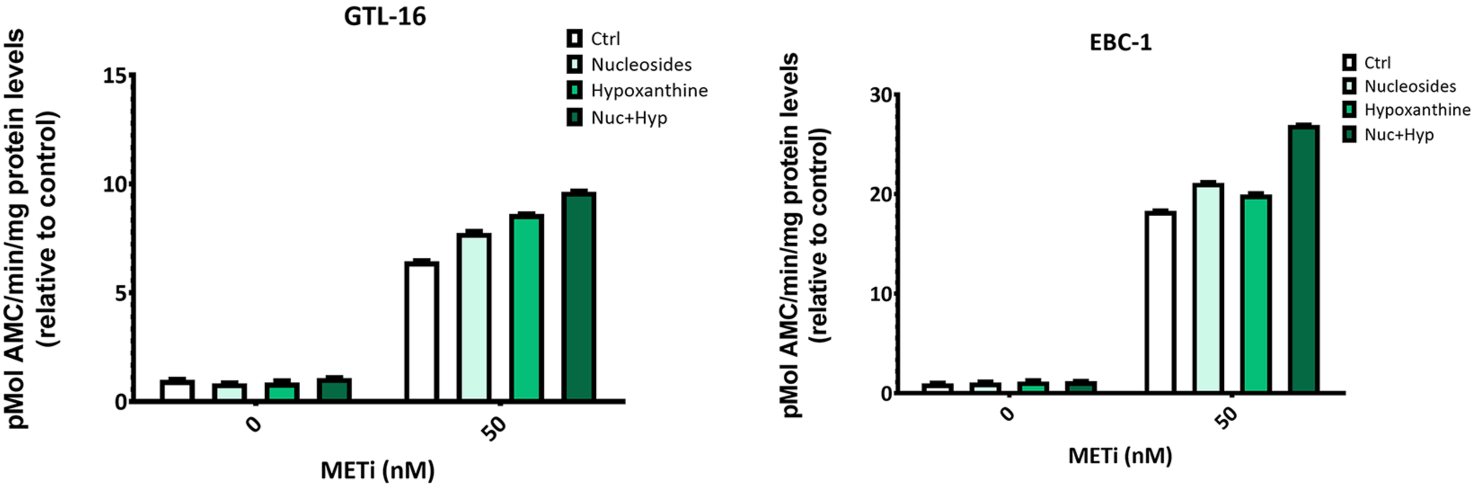

Supplementary figure 4

Supplementary Figure 4: Supplementations with nucleosides and hypoxanthine

A. Proliferation of GTL-16 and EBC-1 cells upon supplementation with nucleosides or hypoxanthine.
B. Apoptosis (caspase-3 activation) in GTL-16 and EBC-1 upon supplementation with nucleosides, hypoxanthine, or their combination.
