# Supplementary material for: E2F1-Associated Purine Synthesis Pathway Is a Major Component of the MET-DNA Damage Response Network
Source: Cancer Res Commun. 2024 Jul 30;4(7):1863–80. doi: 10.1158/2767-9764.CRC-23-0370 (PMC11288008; doi:10.1158/2767-9764.CRC-23-0370)
Supplement: Figure S5 — GART and E2F1 mRNA levels following METi combined with IR: mRNA levels of GART (left) and E2F1 (right) following METi (50nM, 24 hr), IR (10 Gy, 1 hr) and their combination in GTL-16 and EBC-1 cells. [file crc-23-0370_figure_s5_supps5.pdf]

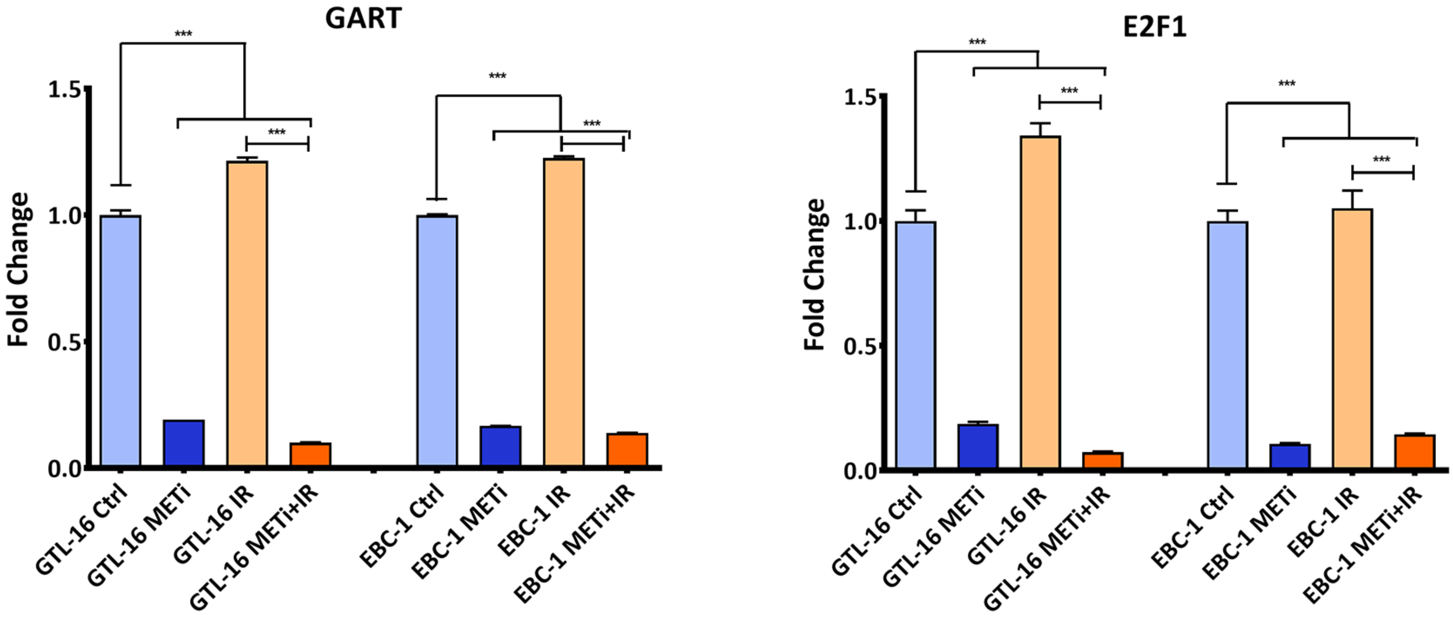

Supplementary figure 5

Supplementary Figure 5: GART and E2F1 mRNA levels following METi combined with IR

mRNA levels of GART (*left*) and E2F1 (*right*) following METi (50nM, 24 hr), IR (10 Gy, 1 hr) and their combination in GTL-16 and EBC-1 cells.
