# Supplementary material for: E2F1-Associated Purine Synthesis Pathway Is a Major Component of the MET-DNA Damage Response Network
Source: Cancer Res Commun. 2024 Jul 30;4(7):1863–80. doi: 10.1158/2767-9764.CRC-23-0370 (PMC11288008; doi:10.1158/2767-9764.CRC-23-0370)
Supplement: Figure S6 — GART protein expression upon MAPK and PI3K targeting: GART protein levels in untreated GTL-16 and EBC-1 cells and after MAPK (AZD6244) and PI3K (LY294002) pathways inhibition. ß Actin was used as a loading control. [file crc-23-0370_figure_s6_supps6.pdf]

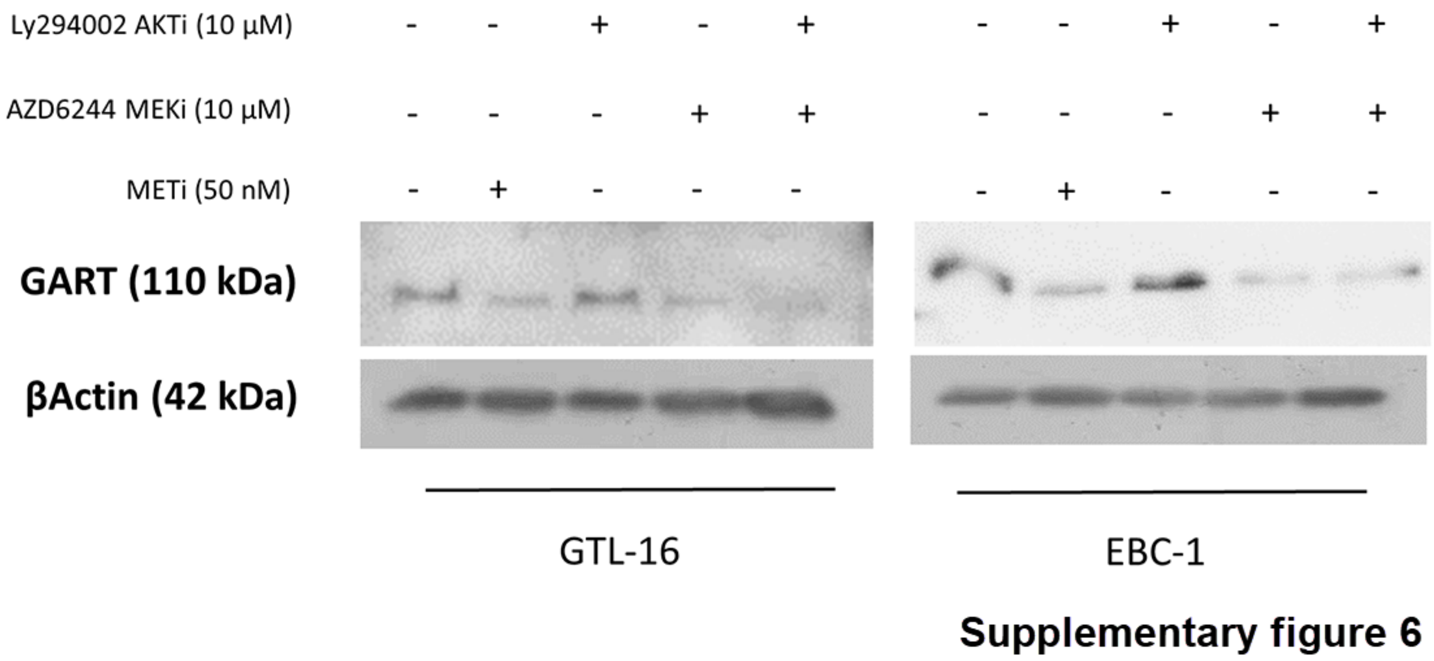

Supplementary Figure 6: GART protein expression upon MAPK and PI3K targeting

GART protein levels in untreated GTL-16 and EBC-1 cells and after MAPK (AZD6244) and PI3K (LY294002) pathways inhibition. ß Actin was used as a loading control.
